# Comparative analysis of differential gene expression analysis tools for single-cell RNA sequencing data

**DOI:** 10.1186/s12859-019-2599-6

**Published:** 2019-01-18

**Authors:** Tianyu Wang, Boyang Li, Craig E. Nelson, Sheida Nabavi

**Affiliations:** 1 Computer Science and Engineering Department, University of Connecticut, Storrs, CT USA; 2 Department of Molecular & Cell Biology, University of Connecticut, Storrs, CT USA; 3 Department of Molecular & Cell Biology, The Institute for Systems Genomics, CLAS, University of Connecticut, Storrs, CT USA; 4 Computer Science and Engineering Department, The Institute for Systems Genomics, University of Connecticut, Storrs, CT USA

**Keywords:** Single-cell, RNAseq, Differential gene expression analysis, Comparative analysis

## Abstract

**Background:**

The analysis of single-cell RNA sequencing (scRNAseq) data plays an important role in understanding the intrinsic and extrinsic cellular processes in biological and biomedical research. One significant effort in this area is the detection of differentially expressed (DE) genes. scRNAseq data, however, are highly heterogeneous and have a large number of zero counts, which introduces challenges in detecting DE genes. Addressing these challenges requires employing new approaches beyond the conventional ones, which are based on a nonzero difference in average expression. Several methods have been developed for differential gene expression analysis of scRNAseq data. To provide guidance on choosing an appropriate tool or developing a new one, it is necessary to evaluate and compare the performance of differential gene expression analysis methods for scRNAseq data.

**Results:**

In this study, we conducted a comprehensive evaluation of the performance of eleven differential gene expression analysis software tools, which are designed for scRNAseq data or can be applied to them. We used simulated and real data to evaluate the accuracy and precision of detection. Using simulated data, we investigated the effect of sample size on the detection accuracy of the tools. Using real data, we examined the agreement among the tools in identifying DE genes, the run time of the tools, and the biological relevance of the detected DE genes.

**Conclusions:**

In general, agreement among the tools in calling DE genes is not high. There is a trade-off between true-positive rates and the precision of calling DE genes. Methods with higher true positive rates tend to show low precision due to their introducing false positives, whereas methods with high precision show low true positive rates due to identifying few DE genes. We observed that current methods designed for scRNAseq data do not tend to show better performance compared to methods designed for bulk RNAseq data. Data multimodality and abundance of zero read counts are the main characteristics of scRNAseq data, which play important roles in the performance of differential gene expression analysis methods and need to be considered in terms of the development of new methods.

**Electronic supplementary material:**

The online version of this article (10.1186/s12859-019-2599-6) contains supplementary material, which is available to authorized users.

## Background

Next generation sequencing (NGS) [1] technologies greatly promote research in genome-wide mRNA expression data. Compared with microarray technologies, NGS provides higher resolution data and more precise measurement of levels of transcripts for studying gene expression. Through downstream analysis of RNA sequencing (RNAseq) data, gene expression levels reveal the variability between different samples. Typically, in RNAseq data analysis, the expression value of a gene from one sample represents the mean of all expression values of the bulk population of cells. Although it is common to use expression values on such a bulk scale in certain situations [2–4], it is not sufficient to employ bulk RNAseq data for other biological research that involves, for example, studying circulating tumor cells [5] and stem cells. Consequently, analyzing gene expression values on the single-cell scale provides deep insight into the interplay between intrinsic cellular processes and stochastic gene expression in biological and biomedical research [6–9]. For example, single-cell data analysis is important in cancer studies, as differential gene expression analysis between different cells can help to uncover driver genes [10].

**Fig. 1:**
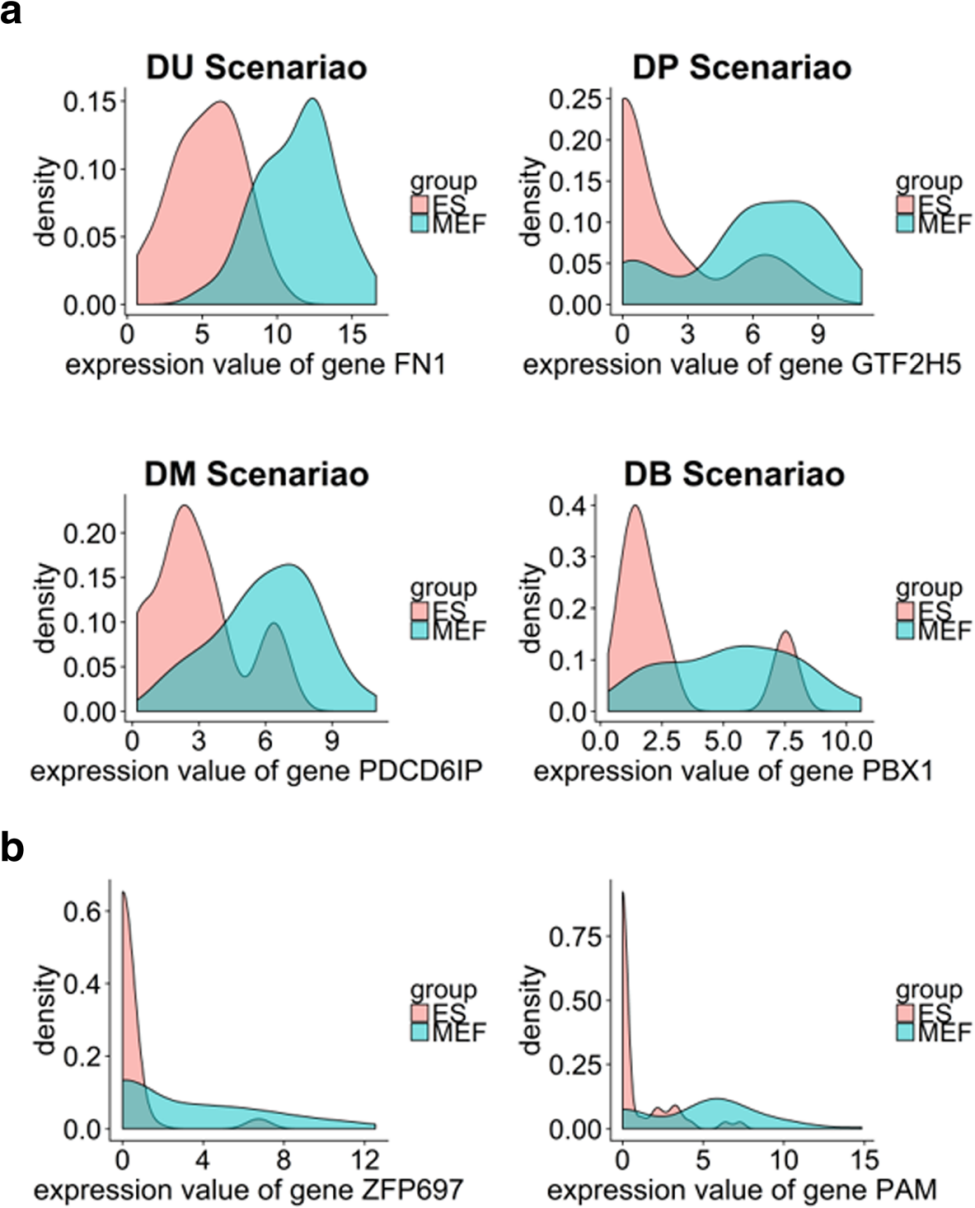
Distributions of gene expression values of total 92 cells in two groups (ES and MEF) using real data show that scRNAseq data exhibit **a** different types of multimodality (DU, DP, DM, and DB) and **b** large amounts of zero counts. X axis represents log-transformed expression values. To clearly show the multimodality of scRNAseq data, zero counts are removed from the distribution plots in (**a**)

Tools developed for differential gene expression analysis on bulk RNAseq data, such as DESeq [11] and edgeR [12], can be applied to single-cell data [11–20]. Single-cell RNAseq (scRNAseq) data, however, have different characteristics from those of bulk RNAseq data that require the use of a new differential expression analysis definition, beyond the conventional definition of a nonzero difference in average expression. In scRNAseq data, due to the tiny number and low capture efficiency of RNA molecules in single cells [6], many transcripts tend to be missed during the reverse transcription. As a result, we may observe that some transcripts are highly expressed in one cell but are missed in another cell. This phenomenon is defined as a “drop-out” event [21]. Recent studies have shown that gene expression in a single cell is a stochastic process and that gene expression values in different cells are heterogeneous [22, 23], which results in multimodality in expression values in different cells. For example, cells from the same brain tissue or the same tumor [24] pose huge heterogeneity from cell to cell [24–28]. Even though they are from the same tissue, these cells are different in regard to cell types, biological functions, and response to drugs. Therefore, unlike bulk RNAseq data, scRNAseq data tend to exhibit an abundance of zero counts, a complicated distribution, and huge heterogeneity. Examples of distributions of scRNAseq expression values between two conditions are shown in Fig. 1. Consequently, the heterogeneity within and between cell populations manifests major challenges to the differential gene expression analysis in scRNAseq data.

To address the challenges of multimodal expression values and/or drop-out events, new strategies and models [21, 29–37] have been proposed for scRNAseq data. Single-cell differential expression (SCDE) [21] and model-based analysis of single-cell transcriptomics **(**MAST) [29] use a two-part joint model to address zero counts; one part corresponds to the normal observed genes, and the other corresponds to the drop-out events. Monocle2 [38] is updated from the previous Monocle [32] and employs census counts rather than normalized transcript counts as input to better normalize the counts and eliminate variability in single-cell experiments. A recent approach, termed scDD [39], considers four different modality situations for gene expression value distributions within and across biological conditions. DEsingle employs a zero-inflated negative binomial (ZINB) regression model to estimate the proportion of the real and drop-out zeros and classifies the differentially expressed (DE) genes into three categories. Recently, nonparametric methods, SigEMD [37], EMDomics [31], and D3E [33], have been proposed for differential gene expression analysis of heterogeneous data. Without modeling the distributions of gene expression values and estimating their parameters, these methods identify DE genes by employing a distance metric between the distributions of genes in two conditions.

A few studies have compared differential expression analysis methods for scRNAseq data. Jaakkola et al. [40] compared five statistical analysis methods for scRNAseq data, three of which are for bulk RNAseq data analysis. Miao et al. [41] evaluated 14 differential expression analysis tools, three of which are newly developed for scRNAseq data and 11 of which are old methods for bulk RNAseq data. A recent comparison study [42] assessed six differential expression analysis tools, four of which were developed for scRNAseq and two of which were designed for bulk RNAseq. In this study, we consider all differential gene expression analysis tools that have been developed for scRNAseq data as of October 2018 (SCDE [21], MAST [29], scDD [39], D3E [33], Monocle2 [38], SINCERA [34], DEsingle [36], and SigEMD [37]). We also consider differential gene expression analysis tools that are designed for heterogeneous expression data (EMDomics [31]) and are commonly used for bulk RNAseq data (edgeR [4], DESeq2 [43]).

The goal of this study is to reveal the limitations of the current tools and to provide insight and guidance in regard to choosing a tool or developing a new one. In this work, we discuss the computational methods used by these tools and comprehensively evaluate and compare the performance of the tools in terms of sensitivity, false discover rate, and precision. We use both simulated and real data to evaluate the performance of the above-noted tools. To generate more realistic simulated data, we model both multimodality and drop-out events in simulated data. Using gold standard DE genes in both simulated and real data, we evaluate the accuracy of detecting true DE genes. In addition, we investigate the agreement among the methods in identifying significantly DE genes. We also evaluate the effect of sample size on the performance of the tools, using simulated data, and compare the runtimes of the tools, using real data. Finally, we perform gene-set enrichment and pathway analysis to evaluate the biological functional relevance of the DE genes identified by each tool.

## Methods

As of October 2018, we have identified eight software tools for differential expression analysis of scRNAseq data, which are designed specifically for such data [21, 29, 30, 33, 34, 36–38] (SCDE, MAST, scDD, D3E, Monocle2, SINCERA, DEsingle, and SigEMD). We also considered tools designed for bulk RNAseq data that are widely used [4, 43] (edgeR, and DESeq2) or can apply to multimodal data [31] (EMDomics). The general characteristics of the eleven tools are provided in Table 1. MAST, scDD, EMDomics, Monocle2, SINCERA, and SigEMD use normalized TPM/FPKM expression values as input, while SCDE, D3E, and DEsingle use read counts obtained from RSEM as input. D3E runs on Python, while all other methods are developed as an R package. In the following sections, we provide the details of the tools.

**Table 1:** Software tools for identifying DE genes using scRNAseq data

Tool	Prog. Language	Input format	Model	Year/ version	URL
SCDE	R	Read counts	Poisson and negative binomial model	2014/2.2.0	http://bioconductor.org/packages/release/bioc/html/scde.html
MAST	R	TPM/FPKM	Generalized linear model	2015/1.0.5	http://bioconductor.org/packages/release/bioc/html/MAST.html
scDD	R	TPM/FPKM	Conjugate Dirichlet process mixture	2016/0.99.0	http://bioconductor.org/packages/devel/bioc/html/scDD.html
EMDomics	R	TPM/FPKM	Non-parametric earth mover’s distance	2016/2.4.0	https://www.bioconductor.org/packages/release/bioc/html/EMDomics.html
D3E	Python	Read counts	Cramér-von Mises test, Kolmogorov-Smirnov test, likelihood ratio test	2016/	https://github.com/hemberg-lab/D3E
Monocle2	R	TPM/FPKM	Generalized additive model	2014/2.2.0	http://bioconductor.org/packages/release/bioc/html/monocle.html
SINCERA	R	TPM/FPKM/Read counts	Welch’s t-test and Wilcoxon rank sum test	2015/	https://research.cchmc.org/pbge/sincera.html
edgeR	R	Read counts	Negative binomial model, Exact test	2010/3.16.5	http://bioconductor.org/packages/release/bioc/html/edgeR.html
DESeq2	R	Read counts	Negative binomial model, Exact test	2014/1.14.1	http://bioconductor.org/packages/release/bioc/html/DESeq2.html
DEsingle	R	Read counts	Zero inflated negative binomial	2018/1.2.0	https://bioconductor.org/packages/release/bioc/html/DEsingle.html
SigEMD	R	TPM/FPKM	Non-parametric earth mover’s distance	2018/0.21.1	https://github.com/NabaviLab/SigEMD

### Differential gene expression analysis methods for scRNAseq data

#### Single-cell differential expression (SCDE)

SCDE [21] utilizes a mixture probabilistic model for gene expression values. The observed read counts of genes are modeled as a mixture of drop-out events by a Poisson distribution and amplification components by a negative binomial (NB) distribution:

$$ \left\{\begin{array}{c}{r}_c\sim NB(e)\ \mathrm{for}\ \mathrm{normal}\ \mathrm{amplified}\ \mathrm{genes}\\ {}{r}_c\sim Possion\left({\lambda}_0\right)\ \mathrm{for}\ \mathrm{drop}-\mathrm{out}\ \mathrm{genes}\end{array}\ \right., $$

where *e* is the expected expression value in cells when the gene is amplified, and *λ*_0_ is always set to 0.1. The posterior probability of a gene expressed at level *x* in cell *c* based on observed *r*_c_ and the fitted model Ω_*c*_ is calculated by:

$$ p\left(x|{r}_c,{\Omega}_c\right)={p}_d(x){p}_{Possion}\left(x|{r}_c\right)+\left(1-{p}_d(x)\right){p}_{NB}\left(x|{r}_c\right), $$

where *p*_*d*_ is the probability of a drop-out event in cell *c* for a gene expressed at an average level *x,* and *p*_*poisson*_(*x*| *r*_c_) and *p*_*NB*_(*x*|*r*_*c*_) are the probabilities of observing expression value *r*_*c*_ in the cases of drop-out (Poisson) and successful amplification (NB) of a gene expressed at level *x* in cell *c,* respectively*.* Then, after the bootstrap step, the posterior probability of a gene expressed at level *x* in a subpopulation of cells *S* is determined as an expected value:

$$ {p}_s(x)=E\left[{\prod}_{c\in B}p\left(x|{r}_c,{\Omega}_c\right)\right], $$

where *B* is the bootstrap samples of *S*. Based on the posterior probabilities of gene expression values in cells *S* and *G*, *p*_*S*_(*x*) and *p*_*G*_(*x*), SCDE uses a fold expression difference *f* in gene *g* for the differential expression analysis between subgroups *S* and *G*, which is determined as:

$$ p(f)={\sum}_{x\in X}{p}_S(x){p}_G(x), $$

where *X* is the expression range of the gene *g*. An empirical *p*-value is determined to test the differential expression.

#### Model-based analysis of single-cell transcriptomics (MAST)

MAST [29] proposes a two-part generalized linear model for differential expression analysis of scRNAseq data. One part models the rate of expression level, using logistic regression:

$$ logit\left(p\left({Z}_{ig}=1\right)\right)={X}_i{\beta}_g^D, $$

where Z = [*Z*_*ig*_] indicates whether gene g is expressed in cell *i*.

The other part models the positive expression mean, using a Gaussian linear model:

$$ p\left({Y}_{ig}=y|{Z}_{ig}=1\right)=N\left({X}_i{\beta}_g^C,{\sigma}_g^2\right), $$

where Y = [*y*_*ig*_] is the expression level of gene *g* in cell *i* observed *Z*_*ig*_ = 1. The cellular detection rate (CDR) for each cell, defined as *CDR*_*i*_ = (1/*N*)∑_*g* = 1_*Z*_*ig*_ (*N* is the total number of genes), is introduced as a column in the design matrix *X*_*i*_ of the logistic regression model and the Gaussian linear model. For the differential expression analysis, a test with asymptotic chi-square null distribution is utilized, and a false discovery rate (FDR) adjustment control [44] is used to decide whether a gene is differentially expressed.

#### Bayesian modeling framework (scDD)

scDD [39] employs a Bayesian modeling framework to identify genes with differential distributions and to classify them into four situations: 1—differential unimodal (DU), 2—differential modality (DM), 3—differential proportion (DP), and 4—both DM and DU (DB), as shown in Additional file 1: Figure S1. The DU situation is one in which each distribution is unimodal but the distributions across the two conditions have different means. The DP situation involves genes with expression values that are bimodally distributed. The bimodal distribution of gene expression values in each condition has two modes with different proportions, but the two modes across the two conditions are the same. DM and DB situations both include genes whose expression values follow a unimodal distribution in one condition but a bimodal distribution in the other condition. The difference is that, in the DM situation, one of the modes of the bimodal distribution is equal to the mode of the unimodal distribution, whereas in the DB situation, there is no common mode across the two distributions.

Let *Y*_*g*_ be the expression value of gene *g* in a collection of cells. The non-zero expression values of gene *g* are modeled as a conjugate Dirichlet process mixture (DPM) model of normals, and the zero expression values of gene *g* are modeled using logistic regression as a separate distributional component:

$$ \left\{\begin{array}{c}\mathrm{nonzero}\ {Y}_g\sim \mathrm{conjugate}\ \mathrm{DPM}\ \mathrm{of}\ \mathrm{normals}\\ {}\mathrm{zero}\ {Y}_g\sim \mathrm{logistic}\ \mathrm{regression}\end{array}\right. $$

For detecting the DE genes, a Bayes factor for gene *g* is determined as:

$$ {BF}_g=\frac{f\left({Y}_g|{M}_{DD}\right)}{f\left({Y}_g|{M}_{ED}\right)}, $$

where *f*(*Y*_*g*_| *M*_*DD*_) is the predictive distribution of the observed expression value from gene *g* under a given hypothesis, *M*_*DD*_ denotes the differential distribution hypothesis, and *M*_*ED*_ denotes the equivalent distribution hypothesis that ignores conditions. As there is no solution for the Bayes factor *BF*_*g*_, a closed form is calculated to present the evidence of whether a gene is differentially expressed:

$$ Scor{e}_g=\log \frac{f\left({Y}_g,{Z}_g|{M}_{DD}\right)}{f\left({Y}_g,{Z}_g|{M}_{ED}\right)}=\log \frac{f_{C1}\left({Y}_g^{C1},{Z}_g^{C1}\right){f}_{C2}Y\left({}_g^{C2},{Z}_g^{C2}\right)}{f_{C1,C2}\left({Y}_g,{Z}_g\right)}, $$

where *Z*_*g*_ is the vector of the mean and the variance for gene *g*, and *C*_*1*_ and *C*_*2*_ represent the two conditions.

### EMDomics

EMDomics [31], a nonparametric method based on Earth Mover’s Distance (EMD), is proposed to reflect the overall difference between two normalized distributions by computing the EMD score for each gene and determining the estimation of FDRs. Suppose *P =* {(*p*_1,_*w*_p1_),(*p*_2,_*w*_p2_)…(*p*_m,_*w*_pm_)} and *Q =* {(*q*_1,_*w*_q1_),(*q*_2,_*w*_q2_)… (*q*_n,_*w*_qn_)} are two signatures, where *p*_*i*_ and *q*_*j*_ are the centers of each histogram bin, and *w*_*pi*_ and *w*_*qj*_ are the weights of each histogram bin. The *COST* is defined as the summation of the multiplication of flow *f*_*ij*_ and the distance *d*_*ij*_:

$$ COST\left(P,Q,F\right)={\sum}_{i=1}^m{\sum}_{j=1}^n{f}_{ij}{d}_{ij}, $$

where *d*_*ij*_ is the Euclidean distance between *p*_*i*_ and *q*_*j*_*,* and *f*_*ij*_ is the amount of weight that need to be moved between *p*_*i*_ and *q*_*j*_. An optimization algorithm is used to find a flow F = [*f*_*ij*_] between *p*_*i*_ and *q*_*j*_ to minimize the *COST*. After that, the EMD score is calculated as the normalized minimum *COST*.

$$ EMD\left(P,Q\right)=\frac{\sum_{i=1}^m{\sum}_{j=1}^n{f}_{ij}{d}_{ij}}{\sum_{i=1}^m{\sum}_{j=1}^n{f}_{ij}} $$

A *q*-value, based on the permutations of FDRs, is introduced to describe the significance of the score for each gene.

### Monocle2

Monocle2 [38] is an updated version of Monocle [32], a computational method used for cell type identification, differential expression analysis, and cell ordering. Monocle applies a generalized additive model, which is a generalized linear method with linear predictors that depend on some smoothing functions. The model relates a univariate response variable *Y*, which belongs to the exponential family, to some predictor variables, as follows:

$$ h\left(E(Y)\right)={\beta}_0+{f}_1\left({x}_1\right)+{f}_2\left({x}_2\right)+\dots +{f}_m\left({x}_m\right), $$

where *h* is the link function, such as identity or log function, *Y* is the gene expression level, *x*_*i*_ is the predictor variable that expresses the cell categorical label, and *f*_*i*_ is a nonparametric function, such as cubic splines or some other smoothing functions. Specifically, the gene expression level *Y* is modeled using a Tobit model:

$$ Y=\left\{\begin{array}{c}{Y}^{\ast }\  if\ {Y}^{\ast }>\lambda \\ {}\lambda\ if\ {Y}^{\ast}\le \lambda \end{array}\right., $$

where Y^*^ is a latent variable that corresponds to predictor *x*, and *λ* is the detection threshold. For identifying DE genes, we use an approximate chi-square (*χ*^2^) likelihood ratio test.

In Monocle2, a census algorithm is used to estimate the relative transcript counts, which leads to an improvement of the accuracy compared with using the normalized read counts, such as TPM values.

#### Discrete distributional differential expression (D3E)

D3E [33] consists of four steps: 1—data filtering and normalization, 2—comparing distributions of gene expression values for DE genes analysis, 3—fitting a Poisson-Beta model, and 4—calculating the changes in parameters between paired samples for each gene. For the normalization, D3E uses the same algorithm as used by DESeq2 [11] and filters genes that are not expressed in any cell. Then, the non-parametric Cramer-von Mises test or the Kolmogorov-Smirnov test is used to compare the expression values’ distributions of each gene for identifying the DE genes. Alternatively, a parametric method, the likelihood ratio test, can be utilized after fitting a Poisson-Beta model:

$$ {\displaystyle \begin{array}{c} PB\left(n|\alpha, \beta, \gamma, \lambda \right)= Poisson\left(n|\frac{\gamma x}{\lambda}\right)\underset{x}{\bigwedge \limits } Beta\left(x|\alpha, \beta \right)\\ {}=\frac{\gamma^n{e}^{-\frac{\gamma }{\lambda }}\varGamma \left(\frac{\alpha }{\lambda }+\frac{\beta }{\lambda}\right)}{\lambda^n\varGamma \left(n+1\right)\varGamma \left(\frac{\alpha }{\lambda }+\frac{\beta }{\lambda }+n\right)\varGamma \left(\frac{\alpha }{\lambda}\right)}\varPhi \left(\frac{\alpha }{\lambda },\frac{\alpha }{\lambda }+\frac{\beta }{\lambda }+n,\frac{\gamma }{\lambda}\right)\end{array}}, $$

where *n* is the number of transcripts of a particular gene, *α* is the rate of promoter activation, *β* is the rate of promoter inactivation, *γ* is the rate of transcription when the promoter is in the active state, *λ* is the transcript degradation rate, and *x* is the auxiliary variable. The parameters *α*, *β*, and *γ* can be estimated by moments matching or Bayesian inference method, but *λ* should be known and assumed to be constant.

### SINCERA

SINCERA [34] is a computational pipeline for single cell downstream analysis that enables pre-processing, normalization, cell type identification, differential expression analysis, gene signature prediction, and key transcription factors identification. SINCERA calculates the *p*-value for each gene from two groups based on a statistical test to identify the DE genes. It provides two methods: one-tailed Welch’s *t*-test for genes, assuming they are from two independent normal distributions, and the Wilcoxon rank sum test for small sample sizes. Last, the FDRs are adjusted, using the Benjamini and Hochberg method [44].

### edgeR

edgeR [4] is a negative binomial model-based method to determine DE genes. It uses a weighted trimmed mean of the log expression ratios to normalize the sequencing depth and gene length between the samples. Then, the expression data are used to fit a negative binomial model, whereby the mean *μ* and variance *ν* have a relationship of *ν  = μ* + *αμ*^*2*^, and *α* is the dispersion factor. To estimate the dispersion factor, edgeR combines a common dispersion across all the genes, estimated by a likelihood function, and a gene-specific dispersion, estimated by the empirical Bayes method. Last, an exact test with FDR control is used to determine DE genes.

### DESeq2

DESeq2 [43] is an advanced version of DESeq [11], which is also based on the negative binomial distribution. Compared with the DESeq, which uses a fixed normalization factor, the new version of DESeq2 allows the use of a gene-specific shrinkage estimation for dispersions. When estimating the dispersion, DESeq2 uses all of the genes with a similar average expression. The fold-change estimation is also employed to avoid identifying genes with small average expression values.

### DEsingle

DEsingle [36] utilizes a ZINB regression model to estimate the proportion of the real and drop-out zeros in the observed expression data. The expression values of each gene in each population of cells are estimated by a ZINB model. The probability mass function (PMF) of the ZINB model for read counts of gene *g* in a group of cells is:

$$ {\displaystyle \begin{array}{c}P\left({N}_g=n|\theta, r,p\right)=\theta \bullet I\left(n=0\right)+\left(1-\theta \right)\bullet {f}_{NB}\left(r,p\right)\\ {}=\theta \bullet I\left(n=0\right)+\left(1-\theta \right)\bullet \left(\begin{array}{c}n+r-1\\ {}n\end{array}\right){p}^n{\left(1-p\right)}^r\end{array}}, $$

where *θ* is the proportion of constant zeros of gene *g* in the group of cells, *I*(*n* = 0) is an indicator function, *f*_*NB*_ is the PMF of the NB distribution, *r* is the size parameter and *p* is the probability parameter of the NB distribution. By testing the parameters (*θ*, *r*, and *p*) of two ZINB models for the two different groups of cells, the method can classify the DE genes into three categories: 1—different expression status (DEs), 2—differential expression abundance (DEa), and 3—general differential expression (DEg). DEs represents genes that they show significant different proportion of cells with real zeros in different groups (i.e. *θ*s are significantly different) but the expression of these genes in the remaining cells show no significance (i.e. *r*, and *p* show no significance). DEa represents genes that they show no significance in the proportion of real zeros, but show significant differential expression in remaining cells. DEg represents genes that they not only have significant difference in the proportion of real zeros, but also significantly expressed differentially in the remaining cells.

### SigEMD

SigEMD [37] employs logistic regression to identify the genes that their zero counts significantly affect the distribution of expression values; and employs Lasso regression to impute the zero counts of the identified genes. Then, for these identified genes, SigEMD employs EMD, similar to EMDomics, for differential analysis of expression values’ distributions including the zero values; while for the remaining genes, it employs EMD for differential analysis of expression values’ distributions ignoring the zero values. The regression model and data imputation declines the impact of large amounts of zero counts, and EMD enhances the sensitivity of detecting DE genes from multimodal scRNAseq data.

### Datasets

In this work, we used both simulated and real data to evaluate the performance of the differential expression analysis tools.

### Simulated data

As we do not know exactly the true DE genes in real single-cell data, we used simulated data to compute the sensitivities and specificities of the eleven methods. Data heterogeneity (multimodality) and sparsity (large number of zero counts), which are the main characteristics of scRNAseq data, are modeled in simulated data. First, we generated 10 datasets, including simulated read counts in the form of log-transformed counts, across a two-condition problem by employing a simulation function from the scDD package [30] in R programing language [45]. For each condition, there were 75 single cells with 20,000 genes in each cell. Among the total 20,000 genes, 2000 genes were simulated with differential distributions, and 18,000 genes were simulated as non-DE genes. The 2000 DE genes were equally divided into four groups, corresponding to the DU, DP, DM, and DB scenarios (Additional file 1: Figure S1). Examples of these four situations from the real data are shown in Fig. 1a. From the 18,000 non-DE genes, 9000 genes were generated, using a unimodal NB distribution (EE scenario), and the other 9000 genes were simulated using a bimodal distribution (EP scenario). All of the non-DE genes had the same mode across the two conditions. Then, we simulated drop-out events by introducing large numbers of zero counts. To introduce zero counts, first, we built the cumulative distribution function (CDF) of the percentage of zeros of each gene, using the real data, *F*_*X*_(*x*). Then, in the simulated data for each gene, we randomly selected *c* (*c*~ *F*_*X*_(*x*)) cells from the total cells for half of the genes in each scenario and forced their expression values to zero (10,000 genes in total). Thus, the CDF of the percentage of zeros of each gene is similar between the simulated and real data (Additional file 1: Figure S2). This way, the distribution of the total counts in the simulated data is more similar to real data, which enables us to assess the true positives (TPs) and false positives (FPs) more accurately.

### Real data

We used the real scRNAseq dataset provided by Islam et al. [46] as the positive control dataset to compute TP rates. The datasets consist of 22,928 genes from 48 mouse embryonic stem cells and 44 mouse embryonic fibroblasts. The count matrix is available in the Gene Expression Omnibus (GEO) database with Accession No. GSE29087. To assess TPs, we used the already-published top 1000 DE genes that are validated through qRT-PCR experiments [47] as a gold standard gene set [21, 40, 42].

We also used the dataset from Grün et al. [48] as the negative control dataset to assess FPs. We retrieved 80 pool-and-split samples that were obtained under the same condition from the GEO database with Accession No. GSE54695. By employing random sampling from the 80 samples, we generated 10 datasets to obtain the statistical characteristics of the results. For each generated dataset, we randomly selected 40 out of the 80 cells as one group and considered the remaining 40 cells as the other group [42]. Because all of the samples are under the same condition, there should be no DE genes in these 10 datasets.

In the preprocessing of the real datasets, we filtered out genes that are not expressed in all cells (zero read counts across all cells), and we used log-transformed transcript per millions (TPM) values as the input.

## Results

### Accuracy of identification of DE genes

#### Results from simulated data

**Fig. 2:**
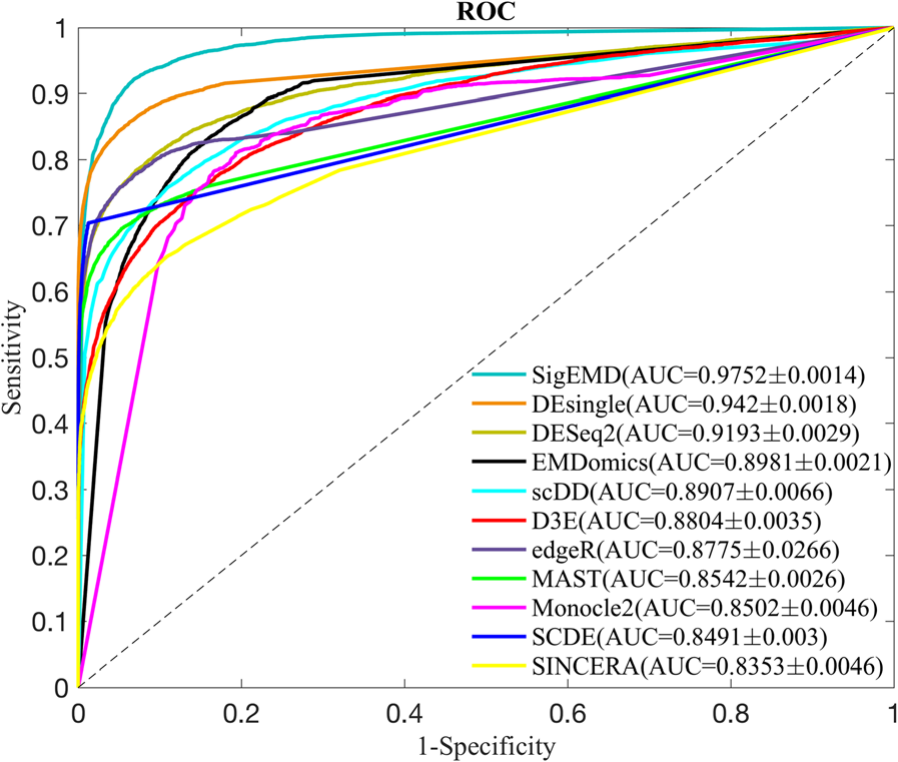
ROC curves for the eleven differential gene expression analysis tools using simulated data

We used simulated data to compute true sensitivities and precision of the tools for detecting DE genes. The receiver operating characteristic (ROC) curves, using the simulated data, are shown in Fig. 2. As can be seen in the figure, the tools show comparable areas-under-the-curve (AUC) values.

The average true positive rates (TPRs, sensitivities), false positive rates (FPRs), precision, accuracy, and F1 score of the tools under the adjusted *p*-value of 0.05 are given in Table 2. We defined TPs as the truly called DE genes, and FPs as the genes that were called significant but were not true DE genes. Similarly, true negatives (TNs) were defined as genes that were not true DE and were not called significant, and false negatives (FNs) were defined as genes that were true DE but were not called significant. We computed TPRs as the number of TPs over the 2000 ground-truth DE genes, FPRs as the number of FPs genes over the 18,000 genes that are not differentially expressed, precision as the number of TPs over all of the detected DE genes, and accuracy as the sum of TPs and TNs over all of the 20,000 genes.

**Table 2:** Numbers of the detected DE genes, sensitivities, false positive rates, precisions, and accuracies of the nine tools using simulated data for an adjusted *p*-value or FDR of 0.05

	Number of detected DE genes	Sensitivity ($\frac{\mathrm{TP}}{\mathrm{TP}+\mathrm{FN}}$)	False positive rate ($\frac{\mathrm{FP}}{\mathrm{FP}+\mathrm{TN}}$)	Precision ($\frac{\mathrm{TP}}{\mathrm{TP}+\mathrm{FP}}$)	Accuracy ($\frac{\mathrm{TP}+ TN}{\mathrm{P}+\mathrm{N}}$)	F1 score ($\frac{2 TP}{2 TP+ FP+ FN}$)
Monocle2	4664.6	0.785	0.172	0.337	0.824	0.472
EMDomics	2465.8	0.666	0.063	0.540	0.910	0.596
DESeq2	2182.6	0.739	0.039	0.677	0.939	0.707
D3E	1683.4	0.565	0.031	0.671	0.929	0.613
scDD	1155.8	0.505	0.008	0.875	0.943	0.640
MAST	954.4	0.470	0.001	0.986	0.946	0.637
edgeR	1161.2	0.557	0.003	0.959	0.953	0.705
SCDE	842	0.419	0.0003	0.994	0.942	0.590
SINCERA	633.6	0.312	0.001	0.984	0.931	0.474
DEsingle	1448.8	0.697	0.003	0.962	0.967	0.808
SigEMD	1456	0.682	0.005	0.937	0.964	0.790

As seen in Table 2, Monocle2 identified the greatest number of true DE genes but also introduced the greatest number of false DE genes, which results in a low identification accuracy, at 0.824. The nonparametric methods, EMDomics and D3E, identified more true DE genes compared to parametric methods (2465.8 and 1683.4 true DE genes, respectively). They also, however, introduced many FPs, resulting in lower accuracies (0.91 and 0.929, respectively) than did parametric methods. In contrast, tools with higher precisions, larger than 0.9 (MAST, SCDE, edgeR, and SINCERA), introduce lower numbers of FPs but identify lower numbers of TPs. Interestingly, F1 scores show that DESeq2 and edgeR, which are designed for traditional bulk RNAseq data, do not show poor performance compared to the tools that are designed for scRNAseq data. DEsingle and SigEMD performed the best in terms of accuracy and F1 score since they identified high TPs and did not introduce many FPs.

**Fig. 3:**
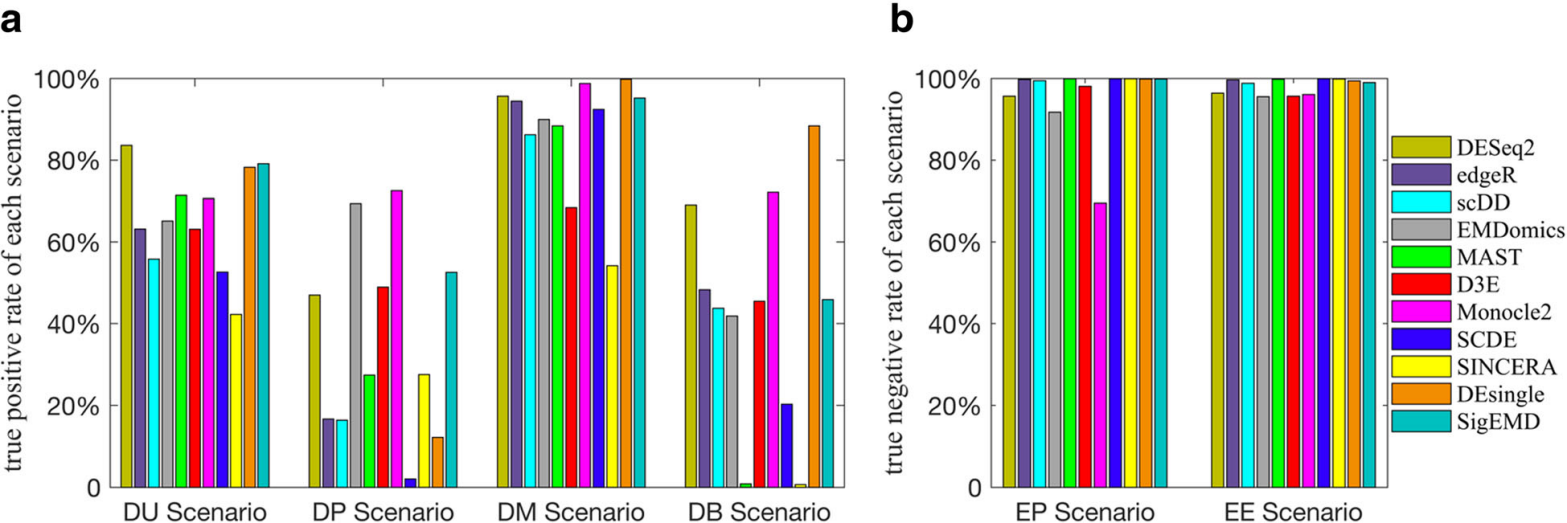
True detection rates for different scenarios of DE genes and non-DE genes using simulated data. **a** true positive rates for DE genes under DU, DP, DM, DB scenarios **b** true negative genes for non-DE genes under EP and EE scenarios

A bar plot of true detection rates of the eleven tools under the four scenarios for DE genes (i.e., DU, DM, DP, and DB) and the two scenarios for non-DE genes (i.e., EP and EE), are shown in Fig. 3. As shown in the figure, all of the methods could achieve a TPR near to or larger than 0.5 for the DU and DM scenarios, where there is no multimodality (DU scenario) or the level of multimodality is low (DM scenario). For scenarios with a high level of multimodality (DP and DB), however, some of the tools, except EMDomics, Monocle2, DESeq2, D3E, DEsingle, and SigEMD, perform poorly. In the DP scenario, only EMDomics and Monocle2 exhibited TPRs larger than 0.5, and SCDE fails for this multimodal scenario. Similarly, for the DB scenario, Monocle2, DESeq2, and DEsingle have a TPR larger than 0.5; however, MAST and SINCERA completely fail. SigEMD exhibited a TPR around 0.5 for both DP and DB scenarios. DEsingle performed the best for the DB scenario but exhibited a low TPR for the DP scenario. We showed the TPRs and true negative rates, using the simulated data with and without large numbers of zeros separately in Additional file 1: Figures S3 and S4. All of the tools have a better performance for the four scenarios when there are not large numbers of zero counts. We also showed the ROC curve for the data with and without large numbers of zeros in Additional file 1: Figures S5 and S6.

It is important to notice that, even though simulated data contain multimodality and zero counts, they cannot capture the real multimodality and zero count behaviors of real data. Therefore, as seen in the following, we evaluated the detection accuracy of detecting DE genes, using real data.

#### Results from positive control real data

We used the positive control real dataset to evaluate the accuracy of the identification of DE genes. We employed the validated 1000 genes as a gold standard gene set. We defined *true detected* DE genes as DE genes that are called by the tools and are among the 1000 gold standard DE genes. The number of detected DE genes and the number of *true detected* DE genes over the 1000 gold standard genes (defined as *sensitivity*) for each tool, using an FDR or adjusted *p*-value of 0.05, are given in Table 3.

**Table 3:** Number of detected DE genes, and sensitivities of the eleven tools using positive control real data for an adjusted *p*-value or FDR of 0.05

	Number of detected DE genes	Sensitivity (TP/1000 gold standard)
Monocle2	8674	0.765
EMDomics	8437	0.762
DESeq2	7612	0.695
D3E	8401	0.722
scDD	2638	0.351
MAST	734	0.198
edgeR	4447	0.58
SCDE	2414	0.392
SINCERA	8366	0.73
DEsingle	9031	0.797
SigEMD	3702	0.488

**Fig. 4:**
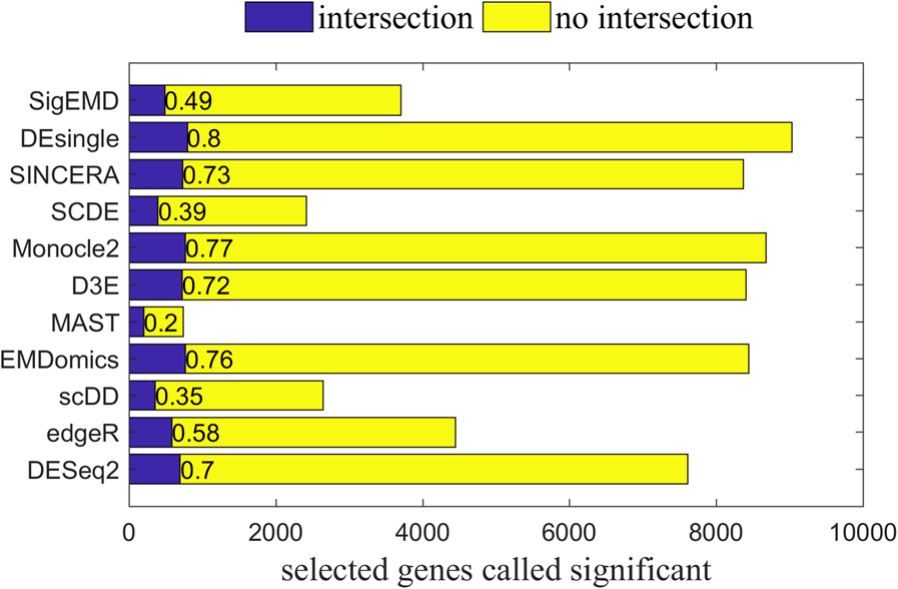
Tools’ total numbers of detected significantly DE genes with the *p*-value or FDR threshold of 0.05 and their overlaps with the 1000 gold standard genes

The tools can be ranked in three levels based on their sensitivities: Monocle2, EMDomics, SINCERA, D3E, and DEsingle rank in the first level, with sensitivities more than 0.7; edgeR, DESeq2, and SigEMD rank in the second level, with sensitivities between 0.4 and 0.7; and SCDE, scDD, and MAST rank in the third level with sensitivities below 0.4. The methods that show better sensitivities, however, also called more than 7000 genes as significantly DE genes. In Fig. 4, the blue bars show the intersection between the gold standard genes and the DE genes called by the methods (*true detected* DE genes), whereas the yellow bars show the number of significantly DE genes that are not among the gold standard genes.

We need to note that we do not have all of the true positive DE genes for the positive control dataset. The 1000 gold standard genes are a subset of DE genes from the dataset that are validated through qRT-PCR experiments [47]. In addition, the datasets that we used in this study have been generated under similar conditions as those of the positive control datasets; however, they are not from the same assay and experiment. Therefore, the results we present here provide information about sensitivities to some degree.

#### Results in negative control real data

Because all of the real true DE genes in the positive control real dataset are unknown, we can test only the TPs, using the 1000 gold standard genes but not the FPs. To validate the FPs, we applied the methods to 10 datasets with two groups, randomly sampled from the negative control real dataset. Because cells in the two groups are from the same condition, we expect the methods to not identify any DE gene. Using an FDR or adjusted *p*-value of 0.05, MAST, SCDE, edgeR, and SINCERA did not call any gene as a DE gene, as we expected, whereas DEsingle, scDD, DESeq2, SigEMD, D3E, EMDomics, and Monocle2 identified 4, 5, 19, 50, 160, 733, and 917 significantly DE genes, respectively, out of 7277 genes in average over the 10 datasets. The number of detected DE genes and FPRs are shown in Table 4. EMDomics and Monocle2, which show the best sensitivities, using the positive control datasets, introduce the most FPs.

**Table 4:** Number of the detected DE genes and false positive rates of the eleven tools using negative control real data for an adjusted *p*-value or FDR of 0.05

	Number of detected DE genes	False positive rate (FP/FP + TN)
Monocle2	917	0.126
EMDomics	733	0.101
DESeq2	19	0.003
D3E	160	0.022
scDD	5	0.0007
MAST	0	0
edgeR	0	0
SCDE	0	0
SINCERA	0	0
DEsingle	4	0.0005
SigEMD	50	0.007

### Agreement among the methods in identifying DE genes

In general, agreement among all of the tools is very low. Considering the top 1000 DE genes detected by the eleven tools in the positive control real data, there are only 92 common DE genes across all of the tools. Of these 92 DE genes, only 41 intersect with the gold standard 1000 DE genes.

**Fig. 5:**
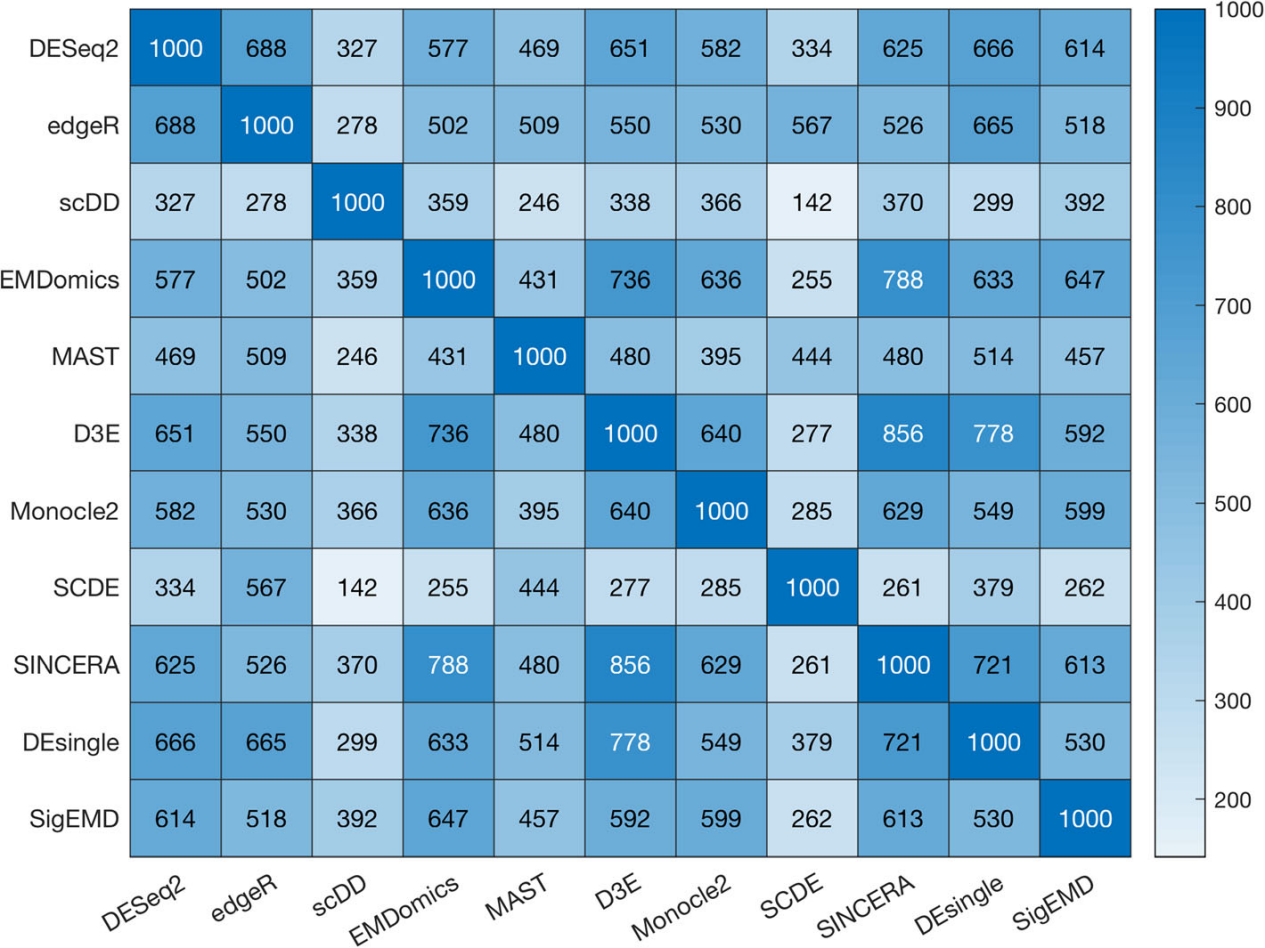
Numbers of pairwise common DE genes tested by top 1000 genes in real data

We investigated how much the tools agreed with each other on identifying DE genes by examining the number of identified DE genes that were common across a pair of tools, which we called *common DE genes*. First, we ranked genes by their adjusted *p*-values or FDRs, and then we selected the top 1000 DE genes. We defined pairwise agreement as the number of *common DE genes* identified by a pair of tools. The numbers of *common DE genes* between pairs of tools are between 770 and 1753 for simulated data (Additional file 1: Figure S7), and 142 and 856 for real data (Fig. 5). We observed that the methods do not have high pairwise agreement in either the simulated data or the real data.

**Fig. 6:**
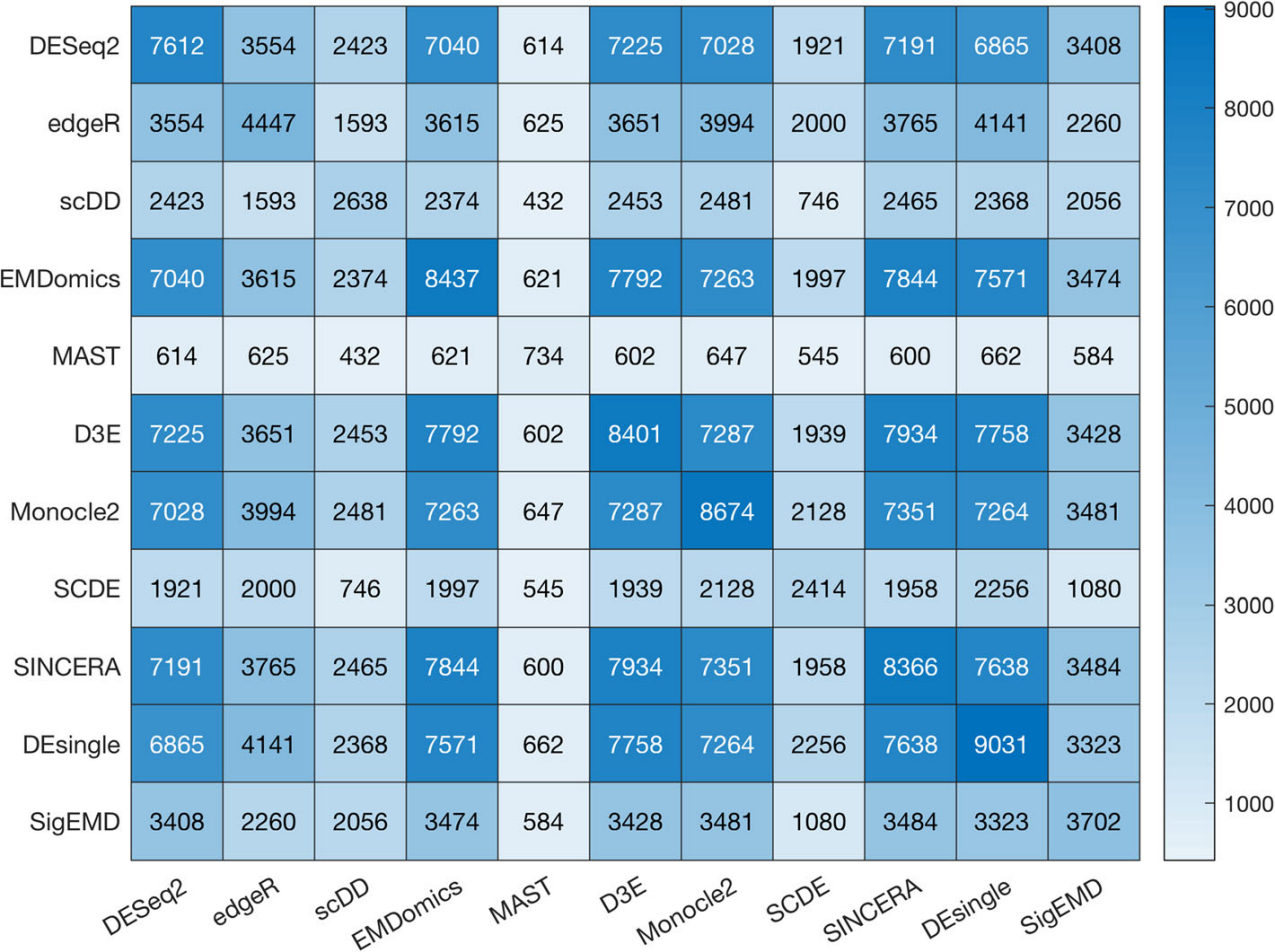
Numbers of pairwise common DE genes tested by adjusted *p*-value< 0.05 in real data

In addition, we used significantly DE genes under a *p*-value or FDR threshold of 0.05 to investigate the pairwise agreement among the tools. The pairwise agreement varies from 432 to 7934 for the real data (Fig. 6) and from 444.8 to 1878 for the simulated data (Additional file 1: Figure S8). In the real data, MAST identified fewer significantly DE genes under the 0.05 cut-off adjusted *p*-value, but the majority of its significantly DE genes overlapped with the significantly DE genes from other tools.

### Effect of sample size

**Fig. 7:**
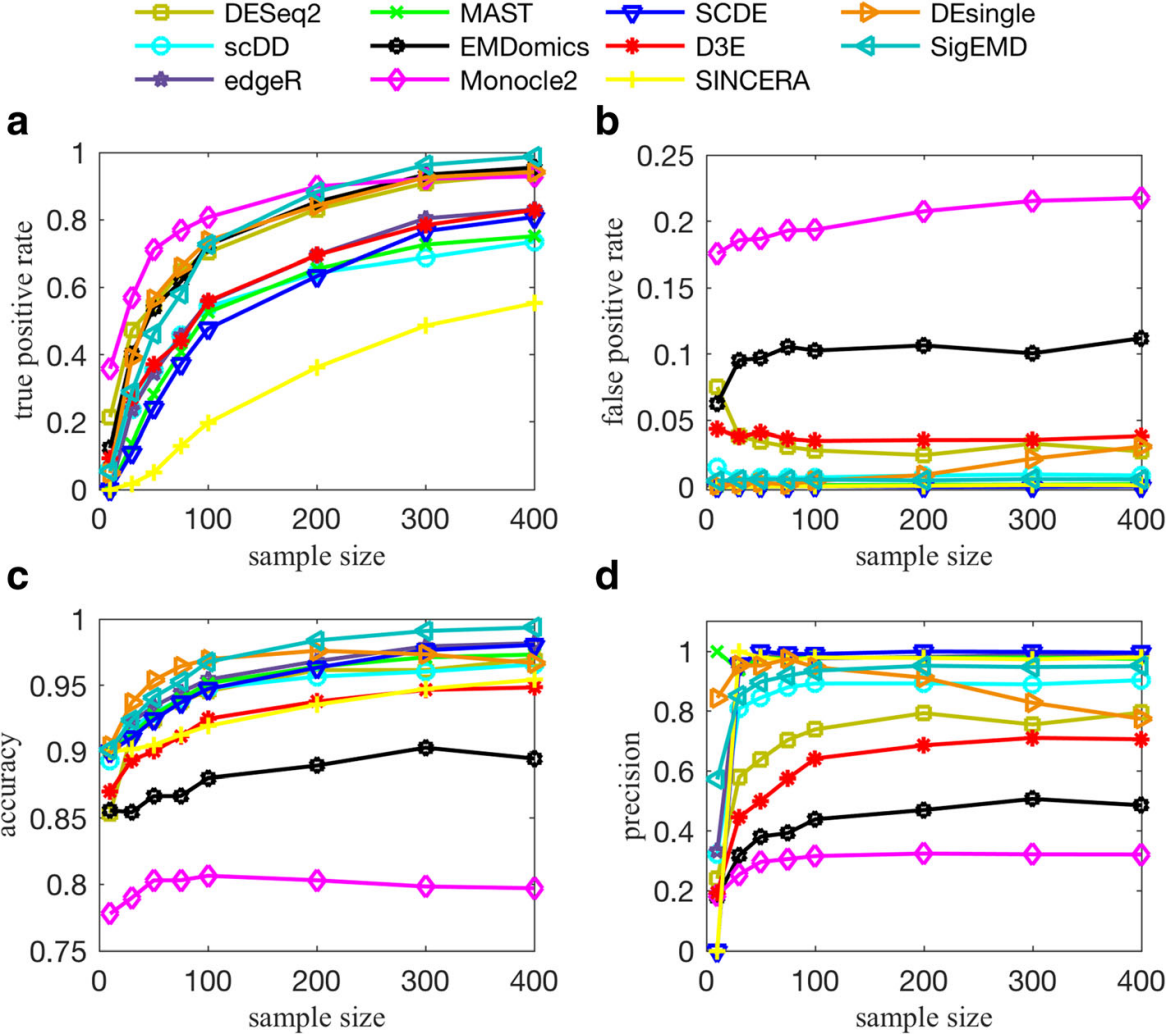
Effect of sample size (number of cells) on detecting DE genes. The sample size is in horizontal axis, from 10 to 400 cells in each condition. Effect of sample size on **a** TPR, **b** FPR, **c** accuracy (=(TP + TN)/(TP + FP + TN + FN)), and precision (=TP/(TP + FP)). A threshold of 0.05 is used for FDR or adjusted *p*-value

We investigated the effect of sample size on detecting DE genes in terms of TPR, FPR, precision, and accuracy, using the simulated data. Precision was defined as TP/(TP + FP) and accuracy as (TP + TN)/(TP + TN + FP + FN). We generated eight cases: 10 cells, 30 cells, 50 cells, 75 cells, 100 cells, 200 cells, 300 cells, and 400 cells for each condition. We noticed that the number of identified DE genes and the TPRs of detection under a default FDR or adjusted *p*-value (< 0.05) tend to increase when the sample size increases from 10 to 400 (Fig. 7) for all tools.

The results show that sample size is very important, as the tools’ precision increases significantly by increasing the sample size from 10 to 75. The FPRs tend to be steady when the sample size is > 75, except for DEsingle. DEsingle works well for a large number of zero counts in a larger dataset. These results also show that Monocle2, EMDomics, DESeq2, DEsingle, and SigEMD can achieve TPRs near 100% by increasing the sample size, while the other methods cannot. Monocle2, EMDomics, DESeq2, and D3E, however, introduce FPs (FPR > 0.05%), whereas FPRs for other methods are very low (close to zero). All of the tools similarly perform poorly for a sample size of < 30. When the sample size exceeded 75 in each condition, the tools achieved better accuracy in detection.

### Enrichment analysis of real data

To examine whether the identified DE genes are meaningful to biological processes, we conducted gene set enrichment analysis through the “Investigate Gene Sets” function of the web-based GSEA software tool (http://www.broadinstitute.org/gsea/msigdb/annotate.js). We investigated the KEGG GENES database (KEGG; contains 186 gene sets) from the Molecular Signatures Database (MSigDB) for the gene set enrichment analysis (FDR threshold of 0.05). We used the same number of identified DE genes (top *n* = 300 genes) of each tool as the input for KEGG pathway enrichment analysis. The results are shown in Table 5. We observed that the 300 top-ranked DE genes identified by nonparametric methods (EMDomics and D3E) were enriched for more KEGG pathways compared to other methods. We also used a box plot to compare the FDRs of the top 10 most significant gene sets enriched by the top-ranked DE genes from the tools (Additional file 1: Figure S9). It can be observed that pathways enriched by the top-ranked DE genes from edgeR and Monocle2 have the highest strength. The 10 top-ranked KEGG pathways for the eleven tools are listed in Additional file 1: Tables S1 to S11.

**Table 5:** Number of KEGG gene sets and GO terms enriched by the top 300 DE genes identified by each tool under an FDR threshold of 0.05

Methods	KEGG	GO Term
EMDomics	53	19
MAST	10	5
D3E	49	10
SCDE	21	9
Monocle2	42	24
SINCERA	39	16
scDD	26	1
DESeq2	39	19
edgeR	39	17
SigEMD	23	15
DEsingle	41	21

We also used DAVID (https://david.ncifcrf.gov/summary.jsp) for the Gene Ontology Process enrichment analysis of the 300 top-ranked DE genes identified by each tool. The numbers of gene ontology (GO) terms under a cutoff FDR of 0.05 are shown in Table 5. Top DE genes identified by EMDomics, D3E, Monocle, and DESeq2 are enriched in more KEGG pathways and/or GO terms compared to those of other tools.

Finally, although the quantitative values of terms recovered from gene set enrichment analysis is informative with regard to the relative statistical power of calling biologically meaningful genes of these tools, very different gene lists can result in very similar quantitative performance values. To perform a qualitative assessment of the biological relevance of the differentially expressed gene lists recovered by each tool, we ranked the performance of each tool in recovering stem cell-relevant GO terms from the 300 top-ranked DE genes. Each gene list was subjected to gene set enrichment against the Biological Process portion of the Gene Ontology Process, and all significant enriched terms were recovered. The results of the Gene Ontology Process enrichment analysis of the 300 top-ranked DE genes and the list of the 300 top-ranked genes for each tool are given in Additional file 2. Significant GO terms with their negative log transform of their *q*-values for each tool are given in Additional file 3. To consolidate closely related processes recovered in this step, we subjected each list of GO terms to word and phrase significance analysis, using world cloud analysis, whereby negative log transform *q*-values are considered as frequencies in this analysis. The phrase significance of each tool, in the form of word clouds, is shown in Additional file 1: Figures S10–S20, and the word significance, in the form of word clouds, is shown in Additional file 1: Figures S21–S31. In these plots, the font size represents the significance of the word/phrase. This provides a readily interpretable visualization of the biologically relevant GO terms.

Several stem cell biologists were then asked to rank the performance of each algorithm in terms of its ability to recover the GO terms most relevant to the experiment that provides the real dataset used in this study. Each algorithm was scored on a 1–3 scale, with 3 as the best recovery of biologically relevant terms and phrases; then, the scores for terms and phrases were added to give an overall performance score from 2 to 6 (Table 6). As expected, many of these tools recovered, at high significance, several terms strongly related to stem cell biology, including development, differentiation, morphogenesis, multicellular, and adhesion as well as many others. Interestingly, scDD and SCDE failed to recover stem cell-relevant terms at high significance. Instead, these approaches appeared to yield terms and phrases related to cellular housekeeping processes. Monocle2 and MAST performed the best at recovering stem cell-relevant terms. Following them, EMDomics, DESeq2, D3E, DEsingle, SigEMD, edgeR, SINCERA all performed well. This result strongly suggests not only that the methods used for identifying DE genes may yield non-overlapping and quantitatively different gene sets but that some methods are much better at extracting biologically relevant gene sets from the data.

**Table 6:** Scores from word and phrase significance analysis of each tool to recover biologically relevant terms and phrases

Methods	Score (phrase)	Score (word)	Overall score (word+phrase)
Monocle2	3	3	6
MAST	3	3	6
DESeq2	2	3	5
D3E	2	3	5
DEsingle	2	3	5
SigEMD	3	2	5
EMDomics	2	2	4
edgeR	2	2	4
SINCERA	2	2	4
SCDE	1	1	2
scDD	1	1	2

### Runtimes

We compared the runtimes of the eleven tools (Table 7). Except for D3E, which was implemented in Python, all of the tools were implemented in R (Table 1). The runtime was computed using a personal computer, iMac with 3.1GHz CPU and up to 8 gigabytes of memory. The average runtime (of 10 times) of each tool, using the positive control dataset, is shown in Table 7. SINCERA has the lowest time cost because it employs a simple *t*-test. edgeR has the lowest time cost among the model-based and nonparametric methods. MAST, Monocle2, and DESeq2 run fast (less than 5 min), as MAST and Monocle2 use linear regression methods, and DESeq2 uses a binomial model for identifying DE genes. scDD takes longer, as it needs time to classify DE genes into different modalities. The nonparametric method, SigEMD, EMDomics and D3E, take more time compared to the model-based methods because they need to compute the distance between two distributions for each gene. We note that D3E had two running modes: It takes about 40 min when running under the simple mode and about 30 h when running under the more accurate mode.

**Table 7:** Average runtime of identifying DE genes in real data by each tool

Methods	Platform	Time consumption in minutes
DESeq2	R	4.2
edgeR	R	0.41
scDD	R	85.13
EMDomics	R	14.64
MAST	R	1.47
D3E	Python	38.43
Monocle2	R	2.6
SCDE	R	10.39
SINCERA	R	0.3
DEsingle	R	14.97
SigEMD	R	14.86

## Discussion

As shown in Fig. 1, scRNAseq expression data are multimodal, with a high number of zero counts that make differential expression analysis challenging. In this study, we conducted a comprehensive evaluation of the performance of eleven software tools for single cell differential gene expression analysis: SCDE, MAST, scDD, EMDomics, D3E, Monocle2, SINCERA, edgeR, DESeq2, DEsingle, and SigEMD. Using simulated data and real scRNAseq data, we compared the accuracy of the tools in identifying DE genes, agreement among the tools in detecting DE genes, and time consumption of the tools. We also examined the enrichment of the identified DE genes by running pathway analysis and GO analysis for the real data.

### Detection accuracy

In general, the eleven methods behave differently in terms of calling true significantly DE genes. The tools that show higher sensitivity also show lower precision. Among all of the tools, DEsingle and SigEMD, which are designed for the scRNAseq, tend to show a better trade-off between TPRs and precision.

All of the tools perform well when there is no multimodality or low levels of multimodality. They all also perform better when the sparsity (zero counts) is less. For data with a high level of multimodality, methods that consider the behavior of each individual gene, such as DESeq2, EMDomics, Monocle2, DEsingle, and SigEMD, show better TPRs. This is because EMDomics and SigEMD use a nonparametric method to compute the distance between two distributions and can capture the multimodality; DEsingle models dropout events well by using a zero inflated negative model to estimate the proportion of real and drop-out zeros in the expression value; Monocle2 uses a census algorithm to estimate the relative transcript counts for each gene instead of using normalized read counts, such as TPM values; and DESeq2 uses a gene-specific shrinkage estimation for the dispersions parameter to fit a negative binomial model to the read counts. If the level of multimodality is low, however, SCDE, MAST, and edgeR can provide higher precision.

### Agreement among the methods

The overall agreement in terms of finding DE genes among all of the tools is low. We used the top 1000 DE genes identified by the eleven tools (ranked by *p*-values) and significantly DE genes with a significant threshold of 0.05 to identify the common DE genes across the tools and between pairs of tools. The DE genes identified by DESeq2, EMDomics, D3E, Monocle2, SINCERA, DEsingle, and SigEMD show higher pairwise agreement, whereas the model-based methods, SCDE and scDD, show less pairwise agreement within other tools. No single tool is clearly superior for identifying DE genes, using single cell sequencing datasets. The tools use different methods with different strengths and limitations for calling DE genes. The sequencing data also are very noisy. The methods treat zero counts, multimodality, and noise differently, resulting in low agreement among them. Some tools work well when the drop-out event is not significant and some, when data multimodality is not significant. For instance, scDD aims at characterizing different patterns of differential distributions; however, handling a large number of zero counts in the expression values is a challenging task for this tool.

### Sample size effect

All of the tools perform better when there are more samples in each condition. TPRs improve significantly by increasing sample size from 10 to 75, but they slow down for sample sizes greater than 100; and for sample sizes of 300 and larger, there are almost no changes in TPRs and FPRs. Monocle2, EMDomics, DESeq2, DEsingle, and SigEMD can achieve a TPR close to 100% by increasing the sample size. DEsingle works well for a larger number of zero counts or small number of samples. When the number of zero counts is low and the number of samples is large, its model cannot capture the dropout event well.

### Enrichment analysis

As expected, top-ranked DE genes of many of these tools are enriched for GO terms strongly related to stem cell biology. scDD and SCDE, however, failed to recover stem cell-relevant terms at high significance. Instead, they appeared to yield GO terms related to cellular housekeeping processes. This result suggests that model-based single cell DE analysis methods that do not consider multimodality do not perform well in extracting biologically relevant gene sets from the data.

## Conclusion

In conclusion, the identification of DE genes, using scRNAseq data, remains challenging. Tools developed for scRNAseq data focus on handling zero counts or multimodality but not both. In general, the methods that can capture multimodality (non-parametric methods), perform better than do the model-based methods designed for handling zero counts. However, a model-based method that can model the drop-out events well, can perform better in terms of true positive and false positive. We observed that methods developed specifically for scRNAseq data do not show significantly better performance compared to the methods designed for bulk RNAseq data; and methods that consider behavior of each individual gene (not all genes) in calling DE genes outperform the other tools. The lack of agreement in finding DE genes by these tools and their limitations in detecting true DE genes and biologically relevant gene sets indicate the need for developing more precise methods for differential expression analysis of scRNAseq data. Multimodality, heterogeneity, and sparsity (many zero counts) are the main characteristics of scRNAseq data that all need to be addressed when developing new methods.

## Additional files

Additional file 1: Supplementary materials (Supplementary Tables S1-S11, Supplementary Figures S1-S31). (DOCX 2225 kb)

Additional file 2: Results of the Gene Ontology Process enrichment analysis of the 300 top-ranked DE genes and the list of the 300 top-ranked genes for each tool. (XLSX 115 kb)

Additional file 3: Significant GO terms with their negative log transform of their q-values for each tool. (CSV 24 kb)

## Abbreviations

AUC: Area under curve
DB: Both DM and DU
DM: Differential modality
DP: Differential proportion
DPM: Dirichlet process mixture
DU: Differential unimodal
FDRs: False discovery rates
FN: False negative
FP: False positive
FPR: False positive rate
GAMs: Generalized additive models
NB: Negative binomial
NGS: Next generation sequencing
RNAseq: RNA sequencing
ROC: Receiver operating characteristic
scRNAseq: Single Cell RNA sequencing
TMM: Trimmed mean of M-values
TN: True negative
TNR: True negative rate
TP: True positive
TPM: Transcript per million
TPR: True positive rate
ZINB: Zero-inflated negative binomial
