# Supplementary material for: Comparative analysis of differential gene expression analysis tools for single-cell RNA sequencing data
Source: BMC Bioinformatics. 2019 Jan 18;20:40. doi: 10.1186/s12859-019-2599-6 (PMC6339299; doi:10.1186/s12859-019-2599-6)
Supplement: Supplementary file 1 — Supplementary materials (Supplementary Tables S1-S11, Supplementary Figures S1-S31). (DOCX 2225 kb) [file 12859_2019_2599_MOESM1_ESM.docx]

**Supplementary**

**Figure S1**

Fig. S1 Density plots of log-transformed expression values of DE genes with four types of scenarios defined in Korthauer et al. [39] including a) DU-differential unimodal, b) DP-differential proportion, c) DM-differential modality, and d) DB-both DP and DM across two groups represented by red and black lines.

**Figure S2**

Fig. S2 Cumulative distribution function of the percentage of zero counts of each gene in the simulated data and the positive control real data.

**Figure S3**

**a**

**b**

Fig. S3 The percentage of a) true positive genes b) true negative genes for each scenario in simulated data without large amounts of zero counts.

**Figure S4**

**a**

**b**

Fig. S4 The percentage of a) true positive genes b) true negative genes for each scenario in simulated data with large amounts of zero counts.

**Figure S5**

Fig. S5 ROC curves for the eleven differential gene expression analysis tools using simulated data without large amounts of zero counts.

**Figure S6**

Fig. S6 ROC curves for the eleven differential gene expression analysis tools using simulated data with large amounts of zero counts.

**Figure S7**

Fig. S7 Numbers of common DE genes tested by top 2000 genes in simulated data.

**Figure S8**

Fig. S8 Numbers of common DE genes tested by adjusted P-value<0.05 in simulated data.

**Figure S9**

Fig. S9 Box plot of FDRs for the top 10 KEGG pathways enriched in the 300 top-ranked genes identified by the tools, using GSEA tool.

**Figure S10**


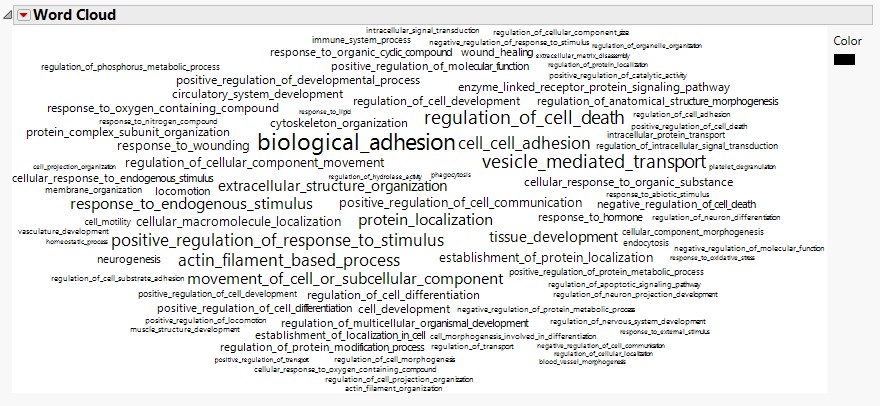


Fig. S10 Phrase cloud using the GO terms enriched by the DE genes identified by D3E.

**Figure S11**


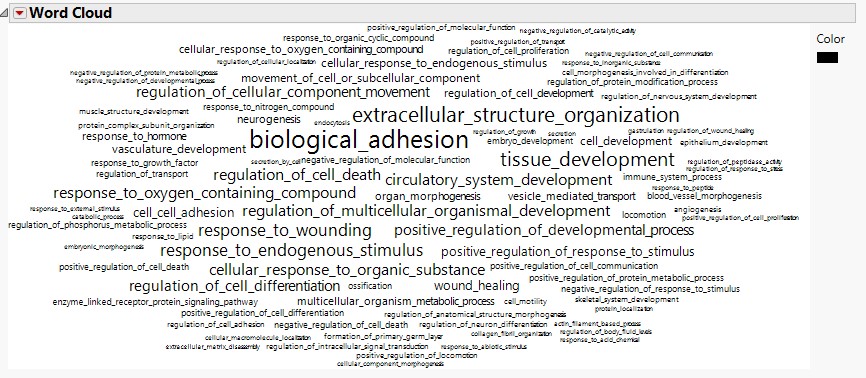


Fig. S11 Phrase cloud using the GO terms enriched by the DE genes identified by DESeq2.

**Figure S12**


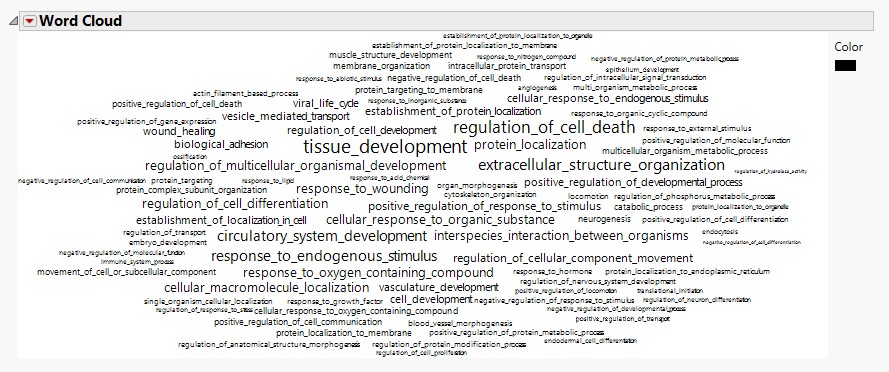


Fig. S12 Phrase cloud using the GO terms enriched by the DE genes identified by edgeR.

**Figure S13**

**
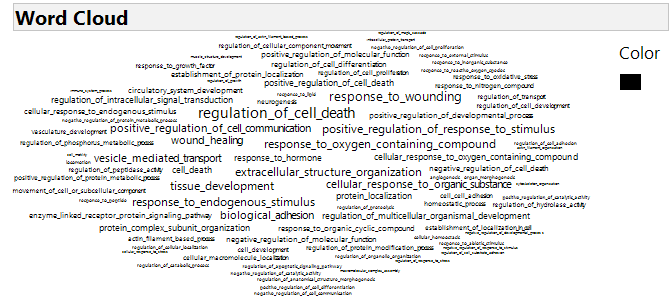
**

Fig. S13 Phrase cloud using the GO terms enriched by the DE genes identified by EMDomics.

**Figure S14**

**
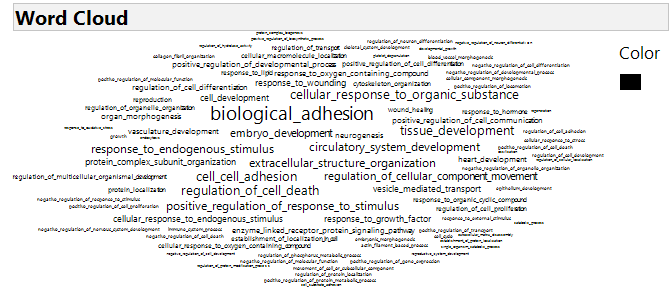
**

Fig. S14 Phrase cloud using the GO terms enriched by the DE genes identified by MAST.

**Figure S15**

**
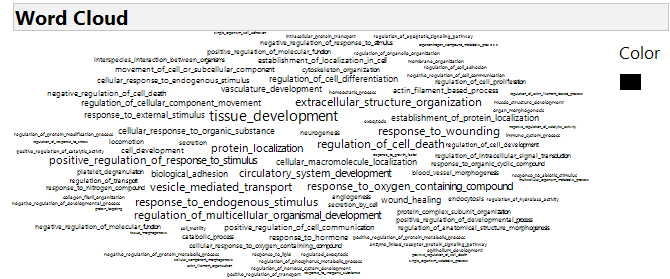
**

Fig. S15 Phrase cloud using the GO terms enriched by the DE genes identified by Monocle2.

**Figure S16**

**
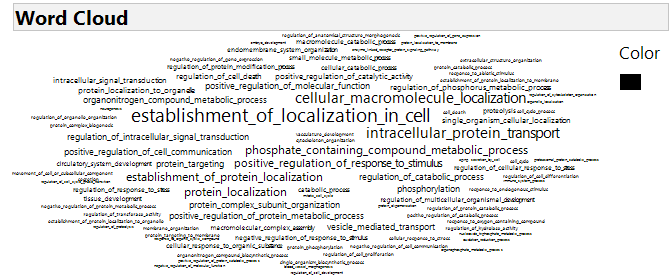
**

Fig. S16 Phrase cloud using the GO terms enriched by the DE genes identified by scDD.

**Figure S17**

**
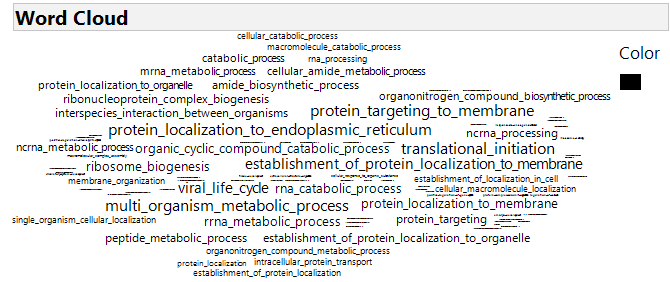
**

Fig. S17 Phrase cloud using the GO terms enriched by the DE genes identified by SCDE.

**Figure S18**


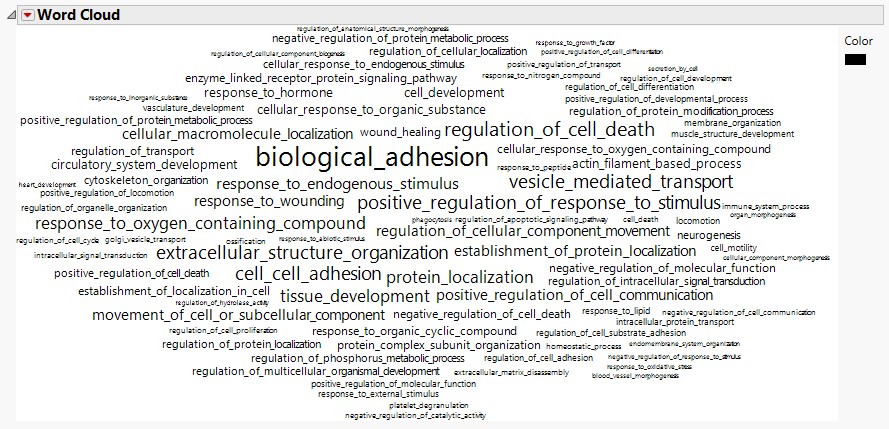


Fig. S18 Phrase cloud using the GO terms enriched by the DE genes identified by SINCERA.

**Figure S19**


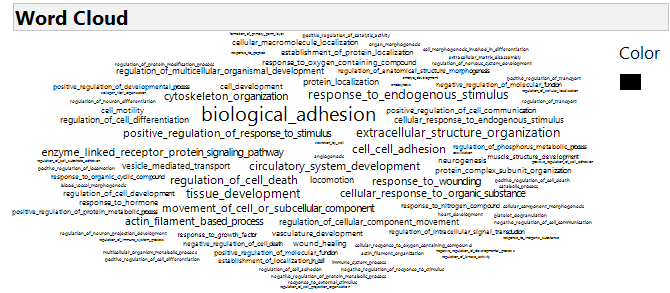


Fig. S19 Phrase cloud using the GO terms enriched by the DE genes identified by DEsingle.

**Figure S20**


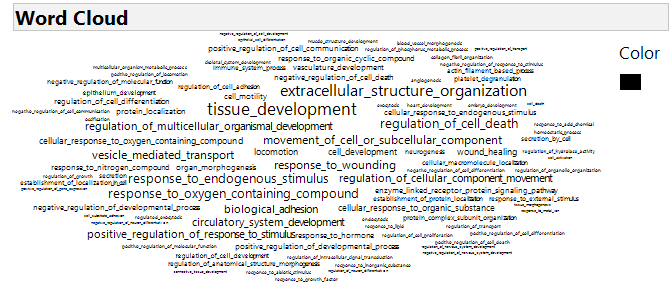


Fig. S20 Phrase cloud using the GO terms enriched by the DE genes identified by SigEMD.

**Figure S21**


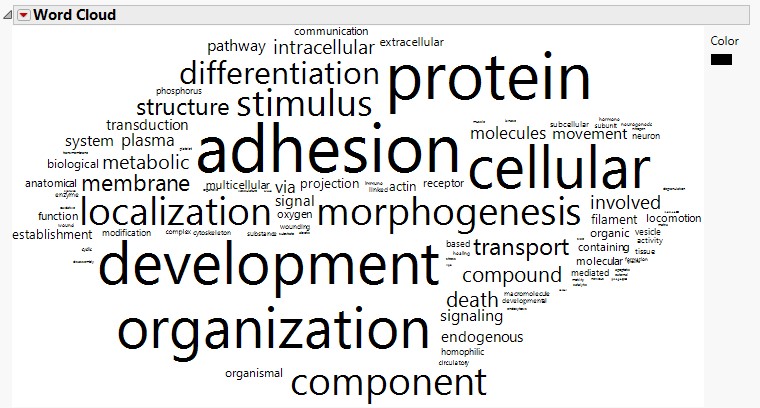


Fig. S21 Word cloud using the GO terms enriched by the DE genes identified by D3E.

**Figure S22**


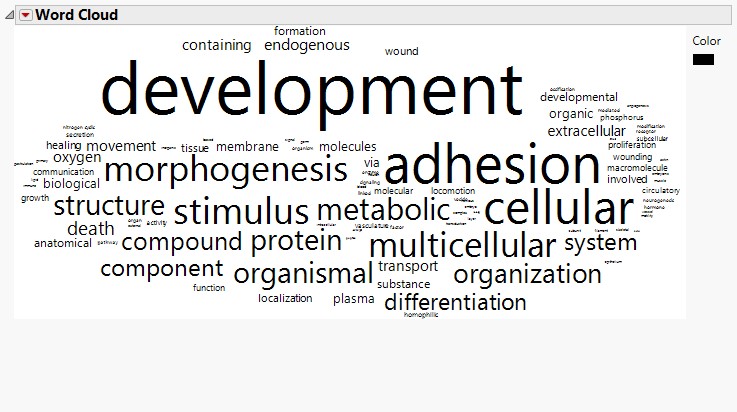


Fig. S22 Word cloud using the GO terms enriched by the DE genes identified by DESeq2.

**Figure S23**


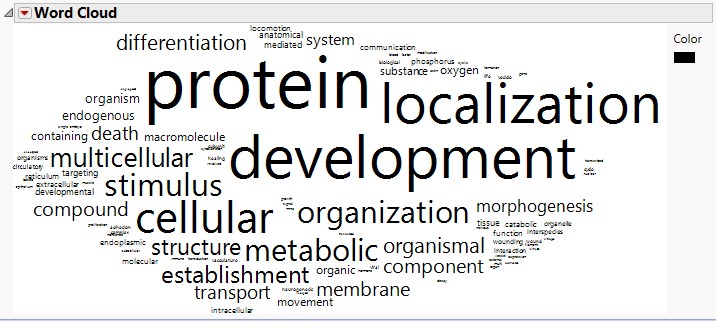


Fig. S23 Word cloud using the GO terms enriched by the DE genes identified by edgeR.

**Figure S24**


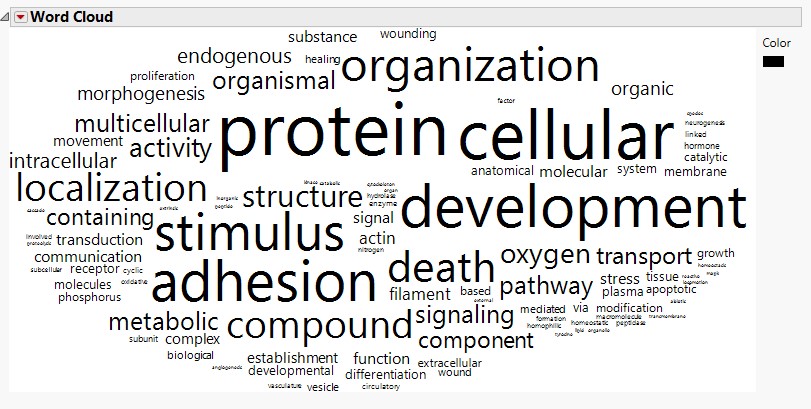


Fig. S24 Word cloud using the GO terms enriched by the DE genes identified by EMDomics.

**Figure S25**


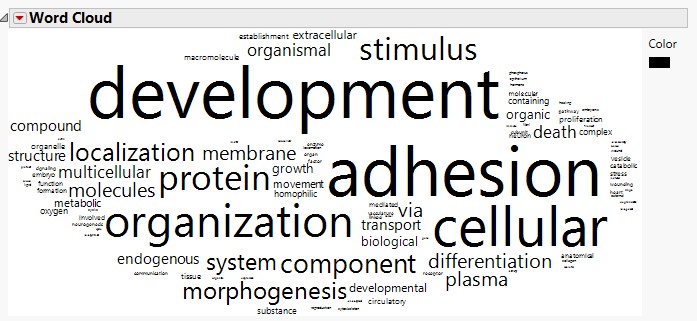


Fig. S25 Word cloud using the GO terms enriched by the DE genes identified by MAST.

**Figure S26**


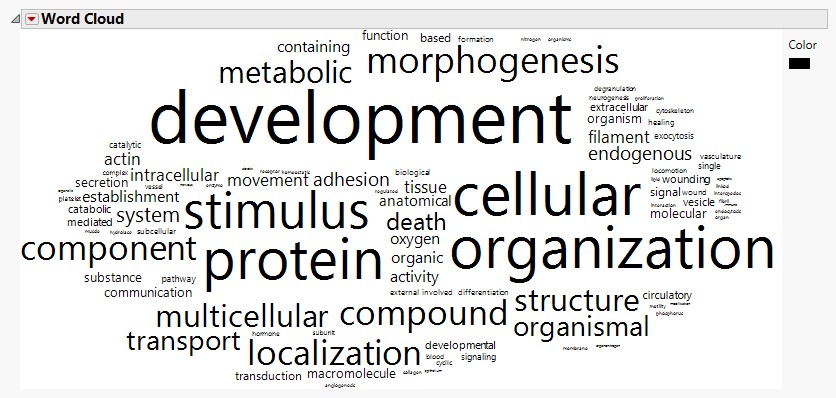


Fig. S26 Word cloud using the GO terms enriched by the DE genes identified by Monocle2.

**Figure S27**


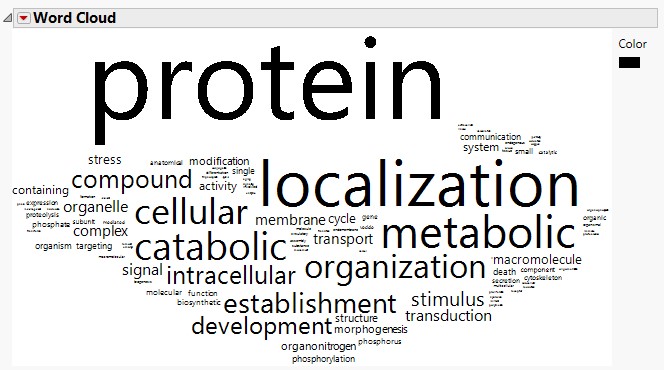


Fig. S27 Word cloud using the GO terms enriched by the DE genes identified by scDD.

**Figure S28**


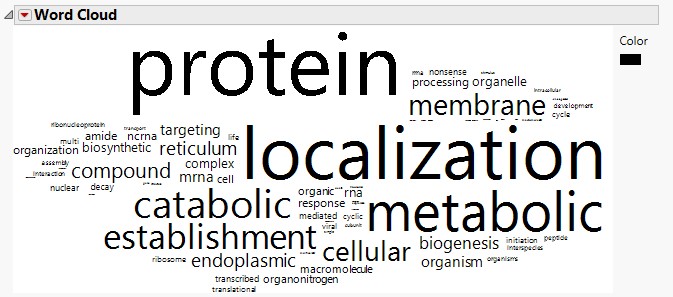


Fig. S28 Word cloud using the GO terms enriched by the DE genes identified by SCDE.

**Figure S29**


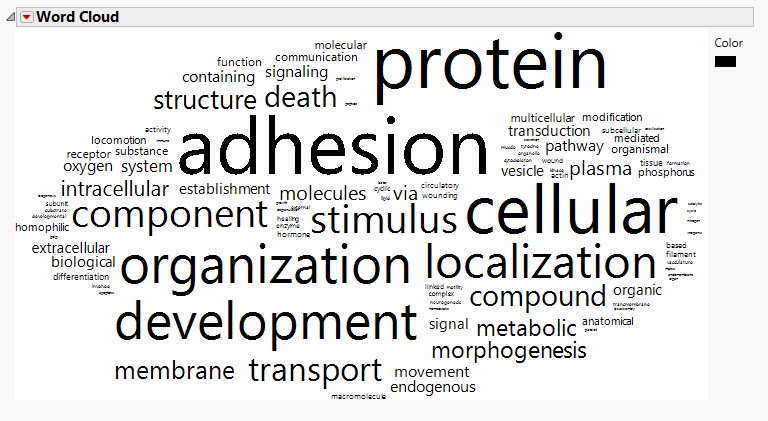


Fig. S29 Word cloud using the GO terms enriched by the DE genes identified by SINCERA.

**Figure S30**

**
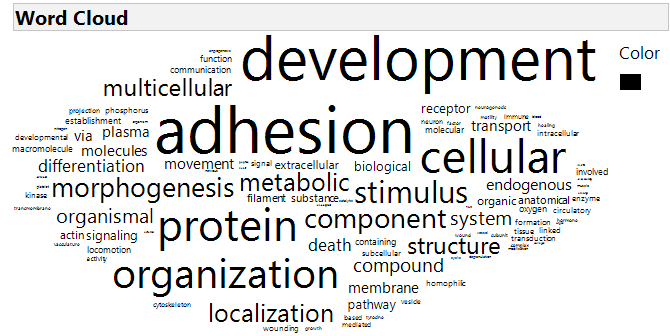
**

Fig. S30 Word cloud using the GO terms enriched by the DE genes identified by DEsingle.

**Figure S31**

**
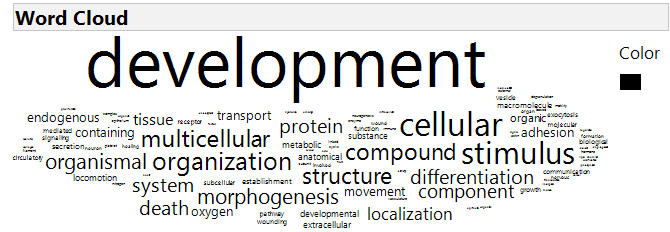
**

Fig. S31 Word cloud using the GO terms enriched by the DE genes identified by SigEMD.

Table S1. Top 10 ranked KEGG pathways enriched by top 300 significant DE genes identified by SCDE

| Gene Set Name | Genes in Gene Set (K) | Description | Genes in Overlap (k) | k/K | FDR q-value |
| --- | --- | --- | --- | --- | --- |
| KEGG_RIBOSOME | 88 | Ribosome | 49 | 0.5568 | 4.31E-89 |
| KEGG_ECM_RECEPTOR_INTERACTION | 84 | ECM-receptor interaction | 8 | 0.0952 | 8.29E-07 |
| KEGG_FOCAL_ADHESION | 201 | Focal adhesion | 9 | 0.0448 | 4.49E-05 |
| KEGG_PATHOGENIC_ESCHERICHIA_COLI_INFECTION | 59 | Pathogenic Escherichia coli infection | 5 | 0.0847 | 4.94E-04 |
| KEGG_BLADDER_CANCER | 42 | Bladder cancer | 4 | 0.0952 | 1.98E-03 |
| KEGG_PATHWAYS_IN_CANCER | 328 | Pathways in cancer | 8 | 0.0244 | 7.01E-03 |
| KEGG_GLYCINE_SERINE_AND_THREONINE_METABOLISM | 31 | Glycine, serine and threonine metabolism | 3 | 0.0968 | 1.23E-02 |
| KEGG_CYSTEINE_AND_METHIONINE_METABOLISM | 34 | Cysteine and methionine metabolism | 3 | 0.0882 | 1.42E-02 |
| KEGG_ANTIGEN_PROCESSING_AND_PRESENTATION | 89 | Antigen processing and presentation | 4 | 0.0449 | 2.01E-02 |
| KEGG_DILATED_CARDIOMYOPATHY | 92 | Dilated cardiomyopathy | 4 | 0.0435 | 2.04E-02 |

Table S2. Top 10 ranked KEGG pathways enriched by top 300 significant DE genes identified by MAST

| Gene Set Name | Genes in Gene Set (K) | Description | Genes in Overlap (k) | k/K | FDR q-value |
| --- | --- | --- | --- | --- | --- |
| KEGG_FOCAL_ADHESION | 201 | Focal adhesion | 15 | 0.0746 | 1.01E-10 |
| KEGG_ECM_RECEPTOR_INTERACTION | 84 | ECM-receptor interaction | 7 | 0.0833 | 4.14E-05 |
| KEGG_LYSOSOME | 121 | Lysosome | 6 | 0.0496 | 3.68E-03 |
| KEGG_REGULATION_OF_ACTIN_CYTOSKELETON | 216 | Regulation of actin cytoskeleton | 7 | 0.0324 | 9.76E-03 |
| KEGG_CELL_CYCLE | 128 | Cell cycle | 5 | 0.0391 | 2.76E-02 |
| KEGG_TIGHT_JUNCTION | 134 | Tight junction | 5 | 0.0373 | 2.82E-02 |
| KEGG_SYSTEMIC_LUPUS_ERYTHEMATOSUS | 140 | Systemic lupus erythematosus | 5 | 0.0357 | 2.94E-02 |
| KEGG_TGF_BETA_SIGNALING_PATHWAY | 86 | TGF-beta signaling pathway | 4 | 0.0465 | 3.11E-02 |
| KEGG_FC_GAMMA_R_MEDIATED_PHAGOCYTOSIS | 97 | Fc gamma R-mediated phagocytosis | 4 | 0.0412 | 4.30E-02 |
| KEGG_ALZHEIMERS_DISEASE | 169 | Alzheimer's disease | 5 | 0.0296 | 4.71E-02 |

Table S3. Top 10 ranked KEGG pathways enriched by top 300 significant DE genes identified by scDD

| Gene Set Name | Genes in Gene Set (K) | Description | Genes in Overlap (k) | k/K | FDR q-value |
| --- | --- | --- | --- | --- | --- |
| KEGG_FOCAL_ADHESION | 201 | Focal adhesion | 13 | 0.0647 | 7.06E-08 |
| KEGG_ALZHEIMERS_DISEASE | 169 | Alzheimer's disease | 12 | 0.071 | 7.06E-08 |
| KEGG_PARKINSONS_DISEASE | 133 | Parkinson's disease | 10 | 0.0752 | 6.23E-07 |
| KEGG_OXIDATIVE_PHOSPHORYLATION | 135 | Oxidative phosphorylation | 10 | 0.0741 | 6.23E-07 |
| KEGG_HUNTINGTONS_DISEASE | 185 | Huntington's disease | 11 | 0.0595 | 9.23E-07 |
| KEGG_PATHWAYS_IN_CANCER | 328 | Pathways in cancer | 13 | 0.0396 | 4.94E-06 |
| KEGG_UBIQUITIN_MEDIATED_PROTEOLYSIS | 138 | Ubiquitin mediated proteolysis | 9 | 0.0652 | 5.71E-06 |
| KEGG_ECM_RECEPTOR_INTERACTION | 84 | ECM-receptor interaction | 6 | 0.0714 | 3.27E-04 |
| KEGG_REGULATION_OF_ACTIN_CYTOSKELETON | 216 | Regulation of actin cytoskeleton | 8 | 0.037 | 1.29E-03 |
| KEGG_P53_SIGNALING_PATHWAY | 69 | p53 signaling pathway | 5 | 0.0725 | 1.29E-03 |

Table S4. Top 10 ranked KEGG pathways enriched by top 300 significant DE genes identified by EMDomics

| Gene Set Name | Genes in Gene Set (K) | Description | Genes in Overlap (k) | k/K | FDR q-value |
| --- | --- | --- | --- | --- | --- |
| KEGG_FOCAL_ADHESION | 201 | Focal adhesion | 21 | 0.1045 | 2.38E-17 |
| KEGG_ECM_RECEPTOR_INTERACTION | 84 | ECM-receptor interaction | 13 | 0.1548 | 6.27E-13 |
| KEGG_REGULATION_OF_ACTIN_CYTOSKELETON | 216 | Regulation of actin cytoskeleton | 14 | 0.0648 | 6.89E-09 |
| KEGG_LYSOSOME | 121 | Lysosome | 10 | 0.0826 | 2.39E-07 |
| KEGG_P53_SIGNALING_PATHWAY | 69 | p53 signaling pathway | 8 | 0.1159 | 4.57E-07 |
| KEGG_FC_GAMMA_R_MEDIATED_PHAGOCYTOSIS | 97 | Fc gamma R-mediated phagocytosis | 8 | 0.0825 | 5.66E-06 |
| KEGG_BLADDER_CANCER | 42 | Bladder cancer | 6 | 0.1429 | 6.37E-06 |
| KEGG_TGF_BETA_SIGNALING_PATHWAY | 86 | TGF-beta signaling pathway | 7 | 0.0814 | 2.56E-05 |
| KEGG_PATHWAYS_IN_CANCER | 328 | Pathways in cancer | 12 | 0.0366 | 2.56E-05 |
| KEGG_AXON_GUIDANCE | 129 | Axon guidance | 8 | 0.062 | 3.02E-05 |

Table S5. Top 10 ranked KEGG pathways enriched by top 300 significant DE genes identified by Monocle2

| Gene Set Name | Genes in Gene Set (K) | Description | Genes in Overlap (k) | k/K | FDR q-value |
| --- | --- | --- | --- | --- | --- |
| KEGG_FOCAL_ADHESION | 201 | Focal adhesion | 23 | 0.1144 | 2.07E-20 |
| KEGG_ECM_RECEPTOR_INTERACTION | 84 | ECM-receptor interaction | 15 | 0.1786 | 2.93E-16 |
| KEGG_REGULATION_OF_ACTIN_CYTOSKELETON | 216 | Regulation of actin cytoskeleton | 20 | 0.0926 | 3.05E-16 |
| KEGG_LYSOSOME | 121 | Lysosome | 12 | 0.0992 | 5.06E-10 |
| KEGG_LEUKOCYTE_TRANSENDOTHELIAL_MIGRATION | 118 | Leukocyte transendothelial migration | 11 | 0.0932 | 5.83E-09 |
| KEGG_P53_SIGNALING_PATHWAY | 69 | p53 signaling pathway | 9 | 0.1304 | 1.11E-08 |
| KEGG_PATHOGENIC_ESCHERICHIA_COLI_INFECTION | 59 | Pathogenic Escherichia coli infection | 8 | 0.1356 | 6.72E-08 |
| KEGG_RIBOSOME | 88 | Ribosome | 9 | 0.1023 | 7.59E-08 |
| KEGG_HYPERTROPHIC_CARDIOMYOPATHY_HCM | 85 | Hypertrophic cardiomyopathy (HCM) | 8 | 0.0941 | 9.92E-07 |
| KEGG_ALZHEIMERS_DISEASE | 169 | Alzheimer's disease | 10 | 0.0592 | 1.51E-06 |

Table S6. Top 10 ranked KEGG pathways enriched by top 300 significant DE genes identified by D3E

| Gene Set Name | Genes in Gene Set (K) | Description | Genes in Overlap (k) | k/K | FDR q-value |
| --- | --- | --- | --- | --- | --- |
| KEGG_FOCAL_ADHESION | 201 | Focal adhesion | 25 | 0.1244 | 8.12E-23 |
| KEGG_ECM_RECEPTOR_INTERACTION | 84 | ECM-receptor interaction | 13 | 0.1548 | 5.99E-13 |
| KEGG_TIGHT_JUNCTION | 134 | Tight junction | 13 | 0.097 | 1.92E-10 |
| KEGG_REGULATION_OF_ACTIN_CYTOSKELETON | 216 | Regulation of actin cytoskeleton | 15 | 0.0694 | 3.94E-10 |
| KEGG_PATHWAYS_IN_CANCER | 328 | Pathways in cancer | 15 | 0.0457 | 1.06E-07 |
| KEGG_LYSOSOME | 121 | Lysosome | 10 | 0.0826 | 1.54E-07 |
| KEGG_SMALL_CELL_LUNG_CANCER | 84 | Small cell lung cancer | 8 | 0.0952 | 1.53E-06 |
| KEGG_PATHOGENIC_ESCHERICHIA_COLI_INFECTION | 59 | Pathogenic Escherichia coli infection | 7 | 0.1186 | 1.97E-06 |
| KEGG_AXON_GUIDANCE | 129 | Axon guidance | 9 | 0.0698 | 2.65E-06 |
| KEGG_PROSTATE_CANCER | 89 | Prostate cancer | 7 | 0.0787 | 2.72E-05 |

Table S7. Top 10 ranked KEGG pathways enriched by top 300 significant DE genes identified by SINCERA

| Gene Set Name | Genes in Gene Set (K) | Description | Genes in Overlap (k) | k/K | FDR q-value |
| --- | --- | --- | --- | --- | --- |
| KEGG_FOCAL_ADHESION | 201 | Focal adhesion | 22 | 0.1095 | 1.42E-18 |
| KEGG_ECM_RECEPTOR_INTERACTION | 84 | ECM-receptor interaction | 12 | 0.1429 | 2.14E-11 |
| KEGG_LYSOSOME | 121 | Lysosome | 9 | 0.0744 | 4.34E-06 |
| KEGG_PATHOGENIC_ESCHERICHIA_COLI_INFECTION | 59 | Pathogenic Escherichia coli infection | 7 | 0.1186 | 4.34E-06 |
| KEGG_REGULATION_OF_ACTIN_CYTOSKELETON | 216 | Regulation of actin cytoskeleton | 11 | 0.0509 | 5.53E-06 |
| KEGG_TGF_BETA_SIGNALING_PATHWAY | 86 | TGF-beta signaling pathway | 7 | 0.0814 | 3.94E-05 |
| KEGG_PATHWAYS_IN_CANCER | 328 | Pathways in cancer | 11 | 0.0335 | 2.30E-04 |
| KEGG_AXON_GUIDANCE | 129 | Axon guidance | 7 | 0.0543 | 4.37E-04 |
| KEGG_TIGHT_JUNCTION | 134 | Tight junction | 7 | 0.0522 | 4.95E-04 |
| KEGG_DILATED_CARDIOMYOPATHY | 92 | Dilated cardiomyopathy | 6 | 0.0652 | 4.95E-04 |

Table S8. Top 10 ranked KEGG pathways enriched by top 300 significant DE genes identified by DESeq2

| Gene Set Name | Genes in Gene Set (K) | Description | Genes in Overlap (k) | k/K | FDR q-value |
| --- | --- | --- | --- | --- | --- |
| KEGG_FOCAL_ADHESION | 201 | Focal adhesion | 24 | 0.1194 | 8.50E-22 |
| KEGG_ECM_RECEPTOR_INTERACTION | 84 | ECM-receptor interaction | 16 | 0.1905 | 7.19E-18 |
| KEGG_LYSOSOME | 121 | Lysosome | 9 | 0.0744 | 3.37E-06 |
| KEGG_SMALL_CELL_LUNG_CANCER | 84 | Small cell lung cancer | 7 | 0.0833 | 3.11E-05 |
| KEGG_REGULATION_OF_ACTIN_CYTOSKELETON | 216 | Regulation of actin cytoskeleton | 10 | 0.0463 | 3.11E-05 |
| KEGG_P53_SIGNALING_PATHWAY | 69 | p53 signaling pathway | 6 | 0.087 | 1.18E-04 |
| KEGG_PATHWAYS_IN_CANCER | 328 | Pathways in cancer | 11 | 0.0335 | 1.44E-04 |
| KEGG_AXON_GUIDANCE | 129 | Axon guidance | 7 | 0.0543 | 3.18E-04 |
| KEGG_PROSTATE_CANCER | 89 | Prostate cancer | 6 | 0.0674 | 3.25E-04 |
| KEGG_TIGHT_JUNCTION | 134 | Tight junction | 7 | 0.0522 | 3.25E-04 |

Table S9. Top 10 ranked KEGG pathways enriched by top 300 significant DE genes identified by edgeR

| Gene Set Name | Genes in Gene Set (K) | Description | Genes in Overlap (k) | k/K | FDR q-value |
| --- | --- | --- | --- | --- | --- |
| KEGG_FOCAL_ADHESION | 201 | Focal adhesion | 25 | 0.1244 | 1.79E-24 |
| KEGG_ECM_RECEPTOR_INTERACTION | 84 | ECM-receptor interaction | 16 | 0.1905 | 1.12E-18 |
| KEGG_RIBOSOME | 88 | Ribosome | 15 | 0.1705 | 7.21E-17 |
| KEGG_REGULATION_OF_ACTIN_CYTOSKELETON | 216 | Regulation of actin cytoskeleton | 12 | 0.0556 | 1.13E-07 |
| KEGG_SMALL_CELL_LUNG_CANCER | 84 | Small cell lung cancer | 7 | 0.0833 | 1.33E-05 |
| KEGG_TIGHT_JUNCTION | 134 | Tight junction | 8 | 0.0597 | 2.11E-05 |
| KEGG_PATHWAYS_IN_CANCER | 328 | Pathways in cancer | 11 | 0.0335 | 4.77E-05 |
| KEGG_LYSOSOME | 121 | Lysosome | 7 | 0.0579 | 9.84E-05 |
| KEGG_DILATED_CARDIOMYOPATHY | 92 | Dilated cardiomyopathy | 6 | 0.0652 | 2.17E-04 |
| KEGG_PATHOGENIC_ESCHERICHIA_COLI_INFECTION | 59 | Pathogenic Escherichia coli infection | 5 | 0.0847 | 3.04E-04 |

Table S10. Top 10 ranked KEGG pathways enriched by top 300 significant DE genes identified by DEsingle

| Gene Set Name | Genes in Gene Set (K) | Description | Genes in Overlap (k) | k/K | FDR q-value |
| --- | --- | --- | --- | --- | --- |
| KEGG_FOCAL_ADHESION | 201 | Focal adhesion | 25 | 0.1244 | 1.73E-23 |
| KEGG_ECM_RECEPTOR_INTERACTION | 84 | ECM-receptor interaction | 14 | 0.1667 | 7.67E-15 |
| KEGG_REGULATION_OF_ACTIN_CYTOSKELETON | 216 | Regulation of actin cytoskeleton | 14 | 0.0648 | 2.92E-09 |
| KEGG_LYSOSOME | 121 | Lysosome | 11 | 0.0909 | 7.28E-09 |
| KEGG_PATHOGENIC_ESCHERICHIA_COLI_INFECTION | 59 | Pathogenic Escherichia coli infection | 7 | 0.1186 | 2.08E-06 |
| KEGG_TIGHT_JUNCTION | 134 | Tight junction | 9 | 0.0672 | 3.27E-06 |
| KEGG_AXON_GUIDANCE | 129 | Axon guidance | 8 | 0.062 | 2.65E-05 |
| KEGG_DILATED_CARDIOMYOPATHY | 92 | Dilated cardiomyopathy | 7 | 0.0761 | 2.83E-05 |
| KEGG_PATHWAYS_IN_CANCER | 328 | Pathways in cancer | 11 | 0.0335 | 8.78E-05 |
| KEGG_LEUKOCYTE_TRANSENDOTHELIAL_MIGRATION | 118 | Leukocyte transendothelial migration | 7 | 0.0593 | 1.20E-04 |

Table S11. Top 10 ranked KEGG pathways enriched by top 300 significant DE genes identified by SigEMD

| Gene Set Name | Genes in Gene Set (K) | Description | Genes in Overlap (k) | k/K | FDR q-value |
| --- | --- | --- | --- | --- | --- |
| KEGG_ECM_RECEPTOR_INTERACTION | 84 | ECM-receptor interaction | 16 | 0.1905 | 3.19E-17 |
| KEGG_FOCAL_ADHESION | 201 | Focal adhesion | 20 | 0.0995 | 2.90E-16 |
| KEGG_LYSOSOME | 121 | Lysosome | 12 | 0.0992 | 1.20E-09 |
| KEGG_P53_SIGNALING_PATHWAY | 69 | p53 signaling pathway | 8 | 0.1159 | 5.72E-07 |
| KEGG_REGULATION_OF_ACTIN_CYTOSKELETON | 216 | Regulation of actin cytoskeleton | 12 | 0.0556 | 5.72E-07 |
| KEGG_VASOPRESSIN_REGULATED_WATER_REABSORPTION | 44 | Vasopressin-regulated water reabsorption | 6 | 0.1364 | 1.05E-05 |
| KEGG_PATHOGENIC_ESCHERICHIA_COLI_INFECTION | 59 | Pathogenic Escherichia coli infection | 6 | 0.1017 | 4.67E-05 |
| KEGG_DILATED_CARDIOMYOPATHY | 92 | Dilated cardiomyopathy | 7 | 0.0761 | 4.67E-05 |
| KEGG_SMALL_CELL_LUNG_CANCER | 84 | Small cell lung cancer | 6 | 0.0714 | 3.06E-04 |
| KEGG_HYPERTROPHIC_CARDIOMYOPATHY_HCM | 85 | Hypertrophic cardiomyopathy (HCM) | 6 | 0.0706 | 3.06E-04 |
